# Medically Relevant *Acinetobacter* Species Require a Type II Secretion System and Specific Membrane-Associated Chaperones for the Export of Multiple Substrates and Full Virulence

**DOI:** 10.1371/journal.ppat.1005391

**Published:** 2016-01-14

**Authors:** Christian M. Harding, Rachel L. Kinsella, Lauren D. Palmer, Eric P. Skaar, Mario F. Feldman

**Affiliations:** 1 Department of Molecular Microbiology, Washington University School of Medicine in St. Louis, St. Louis, Missouri, United States of America; 2 Department of Biological Sciences, University of Alberta, Edmonton, Alberta, Canada; 3 Department of Pathology, Microbiology, and Immunology, Vanderbilt University School of Medicine, Nashville, Tennessee, United States of America; McMaster University, CANADA

## Abstract

*Acinetobacter baumannii*, *A*. *nosocomialis*, and *A*. *pittii* have recently emerged as opportunistic human pathogens capable of causing severe human disease; however, the molecular mechanisms employed by *Acinetobacter* to cause disease remain poorly understood. Many pathogenic members of the genus *Acinetobacter* contain genes predicted to encode proteins required for the biogenesis of a type II secretion system (T2SS), which have been shown to mediate virulence in many Gram-negative organisms. Here we demonstrate that *Acinetobacter nosocomialis* strain M2 produces a functional T2SS, which is required for full virulence in both the *Galleria mellonella* and murine pulmonary infection models. Importantly, this is the first *bona fide* secretion system shown to be required for virulence in *Acinetobacter*. Using bioinformatics, proteomics, and mutational analyses, we show that *Acinetobacter* employs its T2SS to export multiple substrates, including the lipases LipA and LipH as well as the protease CpaA. Furthermore, the *Acinetobacter* T2SS, which is found scattered amongst five distinct loci, does not contain a dedicated pseudopilin peptidase, but instead relies on the type IV prepilin peptidase, reinforcing the common ancestry of these two systems. Lastly, two of the three secreted proteins characterized in this study require specific chaperones for secretion. These chaperones contain an N-terminal transmembrane domain, are encoded adjacently to their cognate effector, and their disruption abolishes type II secretion of their cognate effector. Bioinformatic analysis identified putative chaperones located adjacent to multiple previously known type II effectors from several Gram-negative bacteria, which suggests that T2SS chaperones constitute a separate class of membrane-associated chaperones mediating type II secretion.

## Author Summary

Members of the genus *Acinetobacter*, specifically, *A*. *baumannii*, *A*. *pittii*, and *A*. *nosocomialis*, have rapidly emerged as opportunistic human pathogens particularly targeting the immunocompromised patient population. Of significant concern is the fact that many *Acinetobacter-*induced infections are caused by multiply-drug resistant strains severely limiting clinical intervention strategies. In order to best develop new therapeutic treatment options against *Acinetobacter* infections, we first must gain insight into the mechanisms these bacteria utilize to cause disease. One common way bacteria mediate pathogenesis is through the secretion of proteins or toxins. Here we identified and examined the role of a type II secretion system in *Acinetobacter* biology and pathogenesis. We found that *A*. *nosocomialis* secretes multiple proteins through the type II secretion system, including two lipases and a protease. Furthermore, two of the secreted proteins required dedicated membrane-associated chaperones for secretion. These chaperones appear to be present in multiple bacterial species. Lastly, we found that the *Acinetobacter* type II secretion system was required for full virulence in a murine pulmonary infection model, indicating that this secretion system may be used during the course of an *Acinetobacter* infection. Collectively, we have uncovered a new mechanism by which *Acinetobacter* species mediate disease.

## Introduction

Members of the genus *Acinetobacter* are regarded as opportunistic human pathogens of increasing relevance worldwide due in part to the rapid emergence of multiply-drug resistant strains [1]. In fact, the Center for Disease Control and Prevention has recently categorized multi-drug resistant *Acinetobacter* at the serious hazard level, prompting sustained research and action to further prevent its dissemination. Specifically, *A*. *baumannii*, *A*. *pittii*, and *A*. *nosocomialis* of the *Acinetobacter calcoaceticus-baumannii* (*Acb*) complex have become the most medically relevant members of the genus as they are most frequently isolated from health care facilities as well as human tissues [2]. Although *A*. *baumannii* is thought to be the most prevalent and virulent member of the genus *Acinetobacter*, both *A*. *pittii* and *A*. *nosocomialis* are capable of causing severe human disease and are likely under-represented due largely to technological limitations in species identification across clinical laboratories worldwide [3–5].

The ability of *Acinetobacter* to persist in health care facilities has been an active area of investigation; however, it has been mostly limited to the mechanisms utilized to resist antimicrobial therapy, desiccation, and disinfectants. Little is currently known about the virulence factors employed by *Acinetobacter* species (spp.) to colonize and infect different human tissues [6–9]. Recent studies have, however, demonstrated that protein glycosylation [10, 11], capsule production/modulation [12–14], metal acquisition strategies [15, 16], outer membrane proteins [17–19], and alterations in lipid A [8], all contribute to the ability of medically relevant *Acinetobacter* species to cause disease. It has also been shown that *Acinetobacter* spp. produce both type I pili and type IV pili; however, a definitive role for these pili in virulence has not been determined [20–22].

Multiple secretion systems have been identified and characterized for their role in the biology and virulence of medically relevant members of the *Acb*. The most comprehensively studied secretion system in *Acinetobacter* is the type VI secretion system (T6SS), which has been functionally identified and studied in the medically relevant species *A*. *nosocomialis* and *A*. *baumannii*, as well as in the non-pathogenic species *A*. *baylyi* [23, 24]. Recently, it was found that several multidrug resistant strains of *A*. *baumannii* carry a large, self-transmissible plasmid that encodes for the negative regulators of T6SS. It was found that T6SS is silenced in plasmid-containing cells while part of the population loses the plasmid and subsequently activates T6SS [25]. However, unlike *Burkholderia pseudomallei*, which utilizes its T6SS to toxically infect eukaryotic cells [26, 27], the *Acinetobacter* T6SS primarily mediates anti-bacterial killing; yet, a recent study identified the *Acinetobacter* T6SS to be required for full virulence in an insect model [28]. A type V system autotransporter, Ata, has also been characterized and found to mediate biofilm formation, adherence to extracellular matrix proteins, as well as virulence in a murine systemic model of *Acinetobacter* infection [29]. Furthermore, plasmid encoded genes required for the biogenesis of a type IV secretion system (T4SS) in *A*. *baumannii* and *A*. *lwoffii* have been bioinformatically identified [30, 31]; however, no empirical evidence demonstrating their function has been presented. To date, no classical toxins have been described nor have any *bona fide* secretion systems specifically related to disease been discovered in medically relevant *Acinetobacter* members.

Genes encoding proteins predicted to be associated with a type II secretion system (T2SS) have been identified in *A*. *baumannii* [32, 33]. T2SS are multi-protein complexes, evolutionarily related to type IV pili (T4P) systems, which are responsible for the export of proteins from the periplasmic space to the extracellular milieu or to the outer surface of many Gram-negative bacteria [34, 35]. The T2SS is composed of 12–15 proteins comprising four sub-assemblies: a pseudopilus, an inner-membrane platform assembly, an outer-membrane complex, and a secretion ATPase [36]. Effector proteins are first translocated to the periplasm by the general secretory (Sec) pathway or the twin arginine transport (Tat) system, where the targeted proteins can then fold into the correct tertiary and/or quaternary structure prior to association with components of the T2SS [37]. Competently folded effector proteins can then interact with the different subassemblies of the T2SS and be extruded via interactions with the pseudopilus and the outer-membrane secretin [38]. Several Gram-negative pathogens, including *Vibrio cholerae* [39, 40], *Legionella pneumophila* [41, 42], and enterotoxigenic *Escherichia coli* [43], utilize T2SS for the export of toxins as well as proteins associated with the degradation of biopolymers; thus, T2SS can serve both pathogenic and survival roles for bacteria depending on the environmental niche.

Here, utilizing a proteomics approach coupled with mutational analyses, we demonstrate that *Acinetobacter* spp. carry a functional T2SS. We also present the type II secretome of *A*. *nosocomialis* strain M2. Using a mutational analysis approach, we further demonstrated that both the type IV pili system and the T2SS share a common prepilin peptidase, PilD. Importantly, we show that two of the three identified effectors required chaperones for secretion by the T2SS, one of which is a newly characterized protease/chaperone pair. Lastly, we demonstrated that the *Acinetobacter* T2SS contributes to the extracellular lipolytic activity, and the virulence in the both the *Galleria mellonella* infection model and murine pulmonary infection model.

## Results

### Identification of T2SS-associated loci in medically relevant *Acinetobacter* spp

**Fig 1:**
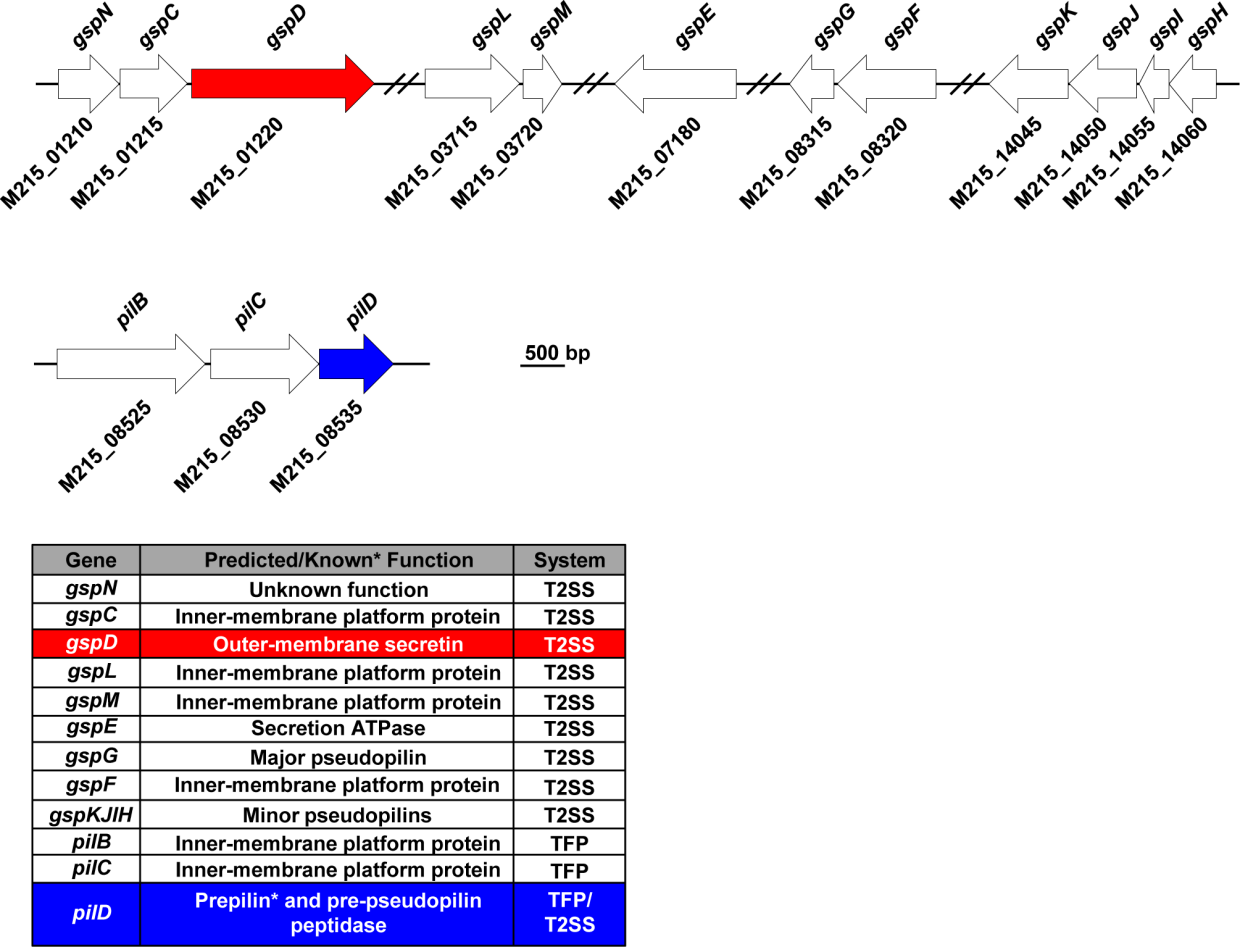
The type II secretion associated gene loci as found in *A*. *nosocomialis* strain M2. Genes predicted to encode proteins required for the biogenesis of a functioning type II secretion system were clustered into five distinct loci and were distantly spread over the chromosome. A single prepilin/pre-pseudopilin peptidase homolog was identified, which was located in the previously described *pilBCD* cluster [45].

Previous manuscripts have reported the bioinformatic identification of genes predicted to encode proteins required for the biogenesis of a T2SS in *Acinetobacter* spp. [32, 33]. We have also identified homologs of genes associated with the biogenesis of a T2SS in *A*. *nosocomialis* strain M2. Here we adopt the *gsp* nomenclature for **g**eneral **s**ecretory **p**athway when defining homologous T2SS associated genes in *Acinetobacter*. Using the Basic Local Alignment Search Tool (BLAST) [44] and homologs of known T2SS-associated genes from *V*. *cholerae*, *P*. *aeruginosa*, and *E*. *coli*, we identified several *gsp* homologs in all publically available genomes from medically relevant *Acinetobacter* spp. Unlike many Gram-negative pathogens encoding a T2SS, the genes encoding predicted type II secretion biogenesis proteins were not encoded in a single operon [35], but were grouped into five distinct gene clusters separated over large distances on the chromosome (Fig 1).

### Differential secretion of proteins in a *gspD-*dependent manner

**Fig 2:**
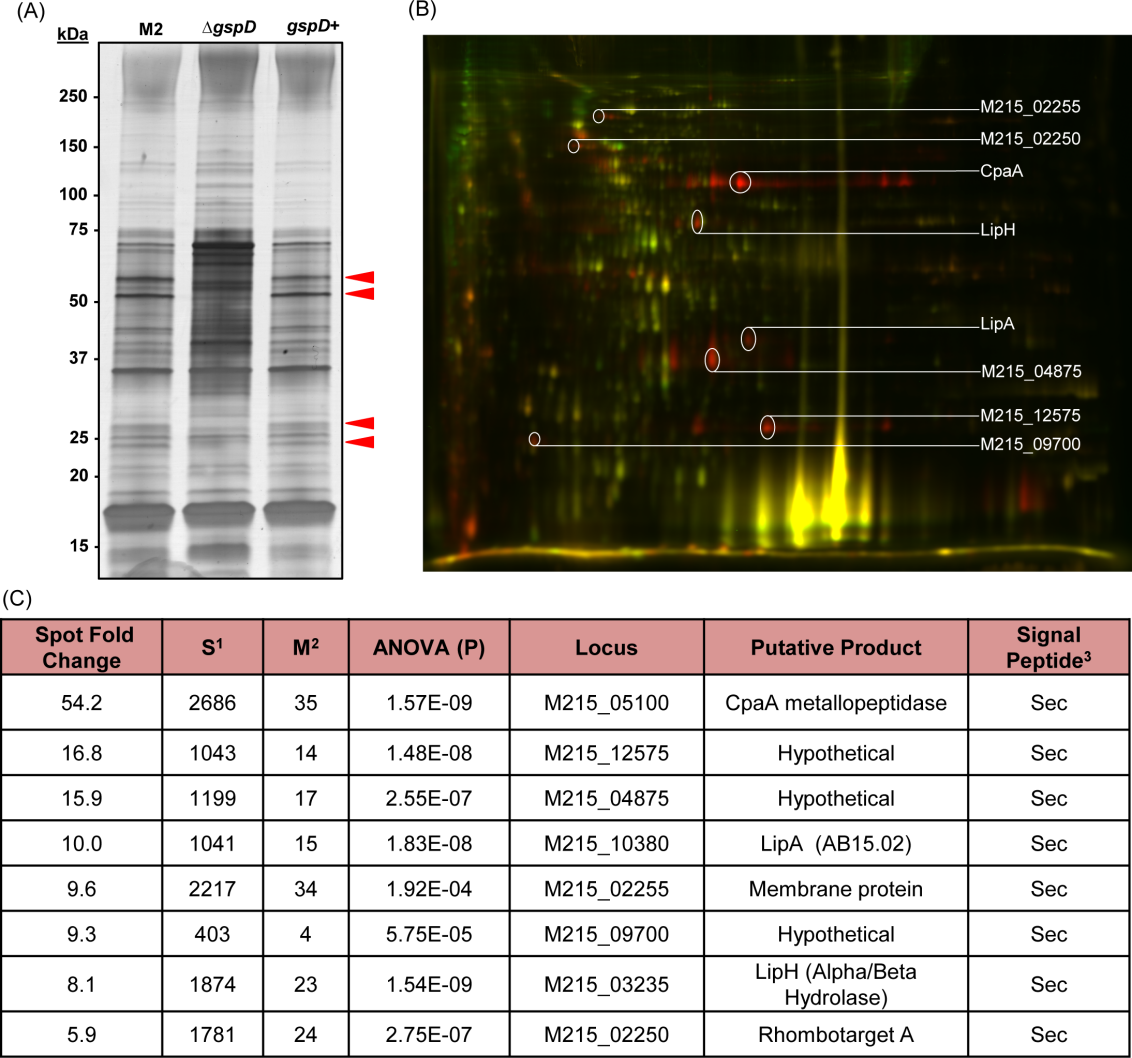
Identification of putative type II secreted proteins from *A*. *nosocomialis* strain M2. (A) Secreted protein fractions from the parent, *∆gspD*::kan mutant, and the *gspD* complemented strain were analyzed by one dimensional SDS-PAGE. Red arrows indicate silver reactive bands that were present in both parent and *gspD* complemented strain’s secreted fractions. (B) Secreted protein fractions from the parent strain and the *∆gspD*::kan mutant were analyzed by two-dimensional difference gel electrophoresis (2D-DIGE). A representative gel image showing Cy3 (Δ*gspD*::kan, green) and Cy5-labeled (parent, red) proteins that were isoelectric focused on pH strips (3–10), separated by size using SDS-PAGE, and visualized using a Typhoon 9400 variable mode imager. A merged image of the Cy3 and Cy5-labeled proteins is shown. Proteins with greater abundance in the Δ*gspD*::kan sample appear green and proteins with greater abundance in the parent strain sample appear red. Proteins that did not change relative abundance between the two samples appear yellow. (C) Putative T2S-dependent proteins identified via 2D-DIGE analyses. Protein candidates associated with the largest spot fold change were bioinformatically examined for the presence of a signal peptide and putative functions. ^1^The protein score is derived from Mascot and provides an indication of how well the peptides matched the indicated protein sequence. The actual score is calculated by the following equation: protein score = -10*Log(P), where P is the probability that the protein match is a random event. Scores above 100 indicate that p < 0.05. ^2^The protein match score indicates the number of unique peptides that matched the sequence of the identified protein. Two unique peptide matches to a protein sequence confirms the identity of a protein. ^1^Signal peptide prediction was bioinformatically predicted using SignalP 4.1.

To test the functionality of the T2SS in *A*. *nosocomialis* strain M2 we deleted the predicted type II outer membrane secretin gene homolog, *gspD*, from strain M2. GspD secretin monomers form a dodecamer complex in the outer-membrane that is required for the export of periplasmic effector proteins (Fig 1) [46, 47]. Using the T2SS deficient M2∆*gspD*::kan mutant we probed for differentially secreted proteins by one-dimensional sodium dodecyl sulfate polyacrylamide gel electrophoresis (SDS-PAGE). Furthermore, we complemented the *gspD*::kan mutant and probed for secreted proteins from this genetic background. The secreted protein profiles from all three strains contained an abundance of proteins; however, differences in the secreted protein profile from the *gspD*::kan mutant were clearly evident when compared to the parental strain. At least 4 silver-reactive protein bands were absent in the secreted profile from the *gspD*::kan mutant when compared to the secreted protein profile from the parental strain (Fig 2A). Importantly, the secreted protein profile from the complemented *gspD* strain showed the same profile as the parental strain M2 indicating that these differences observed in the secreted protein profile from the *gspD*::kan mutant were due to the loss of the putative outer membrane secretin and not to the mutational strategy.

### 2D-DIGE analysis of type II dependent secreted proteins in *A*. *nosocomialis* strain M2

Although our 1D SDS-PAGE analysis strongly indicated that *A*. *nosocomialis* strain M2 did in fact produce a functional T2SS, the abundance of non-type II secreted proteins would interfere with downstream identification. We therefore proceeded with a two-dimensional difference gel electrophoresis (2D-DIGE) analysis to enhance protein separation. The secreted protein fraction from the wild type strain M2 was compared to the secreted protein fraction from the M2*∆gspD*::kan mutant to generate the preliminary type II secretome of *A*. *nosocomialis* strain M2 via 2D-DIGE analysis. Analysis of gel images with SameSpots software (TotalLab, New Castle upon Tyne) revealed that 60 spots exhibited a statistically significant average change of at least 4-fold when comparing wild type M2 vs. M2*∆gspD*::kan samples. A representative gel image from the 2D-DIGE analysis is shown in Fig 2B. Gel spots were cored using an Ettan Spot Handling Workstation and prepared for in gel trypsin digestion. Peptides were eluted and analyzed using capillary-liquid chromatography-nanospray tandem mass spectrometry. The complete list of proteins identified for each spot as well as a detailed description of the 2D-DIGE analysis and methodologies can be found in S1 Appendix; proteins associated with the largest spot fold change, however, are listed in Fig 2C. Three of the proteins identified in Fig 2C, M215_05100, M215_10380, and M215_03235, were of particular interest as all contained domains of known function. The remaining proteins listed in Fig 2C do not contain any known functional domains, with the exception of M215_02250/M215_02255 pair, which was bioinformatically identified as GlyGly-CTERM and rhomobosortase [48].

The top secreted candidate, M215_05100, is an ortholog of the previously identified CpaA metallopeptidase from the M72 family of peptidases, which was proposed to cleave both factor V and fibrinogen [49]. The M72 peptidases are characterized as peptidyl-Asp-endopeptidases containing the HEXXHXXGXX active site, where a zinc ion is predicted to be bound by three histidine residues, and the glutamate is predicted to be the catalytic residue [50]. The M215_10380 locus encodes an ortholog of the previously characterized LipA lipase from *A*. *baylyi* [51, 52], which contains an alpha/beta hydrolase fold from the homologous family abH15.02 (*B*. *cepacia* lipase-like) within the abH15 superfamily (*Burkholderia* lipase superfamily) as determined by the Lipase Engineering Database [53]. These lipases are predicted to have a catalytic triad of a serine, a glutamate or aspartate, and a histidine. Lastly, the M215_03235 locus encodes for another protein containing an alpha/beta hydrolase fold; however, the M215_03235 gene product does not have homology to any know lipases within the Lipase Engineering Database and has yet to be characterized in *Acinetobacter*.

### The prepilin peptidase, PilD, is also the pre-pseudopilin peptidase of the *Acinetobacter* T2SS

**Fig 3:**
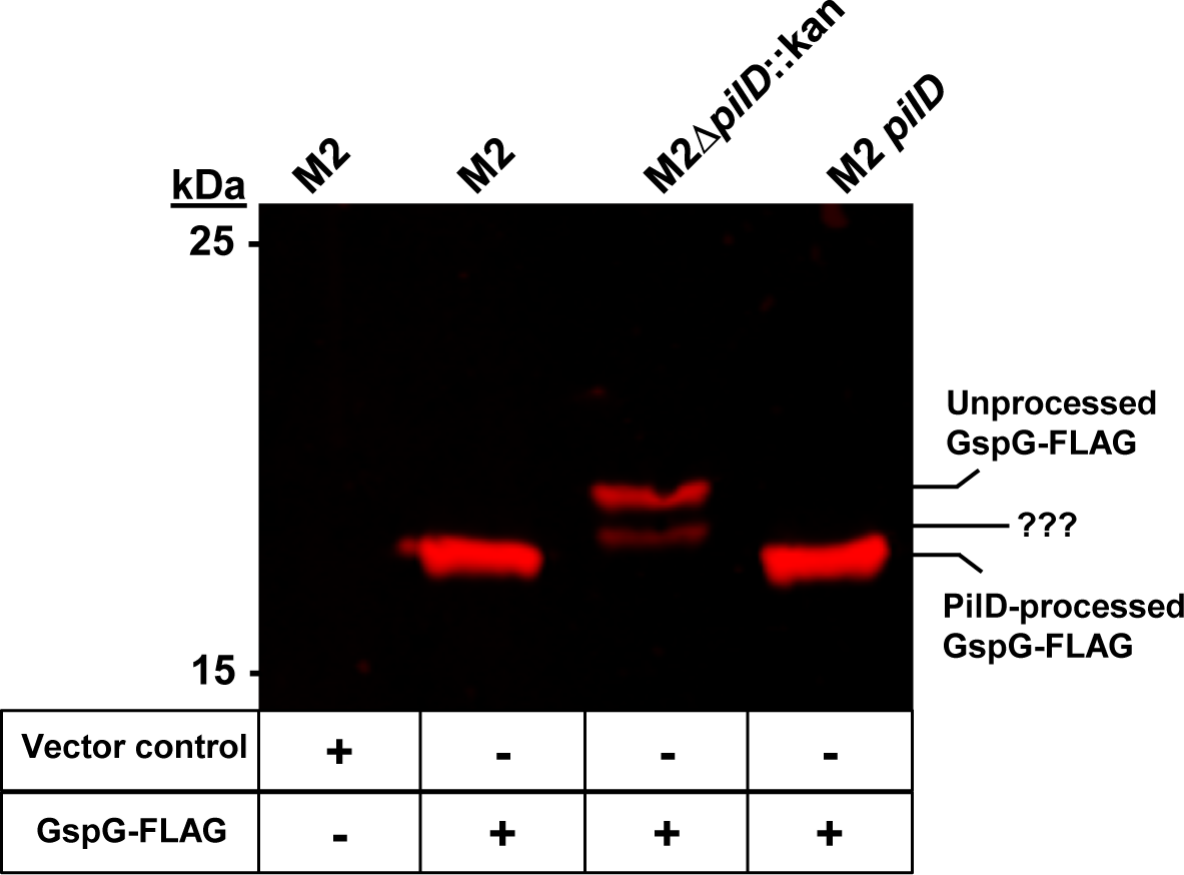
The predicted major pseudopilin, GspG, was processed by the prepilin peptidase PilD. Whole cell lysates from strains containing either the empty vector or pWH-*gspG-FLAG* were examined by western blot and probed for processed and unprocessed GspG-FLAG. GspG-FLAG from both the parent and *pilD* complemented strain migrated at a faster electrophoretic mobility when compared to GspG-FLAG from the *∆pilD*::kan mutant. The theoretical molecular mass of full length GspG and processed GspG is 18,549 Daltons and 14,360 Daltons, respectively.

BLAST analysis revealed the presence of only a single prepilin peptidase, *gspO*/*pilD*, which was previously designated PilD and reported to be the major prepilin peptidase for the type IV pili (T4P) system in *Acinetobacter* (Fig 1) [45]. Given that only one *gspO*/*pilD* homolog was identified in strain M2’s genome as well as in *A*. *baumannii* ATCC 17978 and 19606, we hypothesized that the previously identified prepilin peptidase, PilD, was also the pre-pseudopilin peptidase required for the T2SS. To this end, we cloned and heterologously expressed the predicted major pseudopilin, *gspG*, with a carboxy-terminal FLAG tag in the wild type M2 background, the *∆pilD*::kan mutant, and its respective complement in order to probe for pseudopilin processing. As expected GspG-FLAG expression was detected in all three backgrounds; however, GspG-FLAG from both the wild type M2 and the complemented *pilD* strain migrated with an increased electrophoretic mobility as compared to GspG-FLAG from the *∆pilD*::kan strain (Fig 3). The increase in electrophoretic mobility was most likely due to the loss of the leader sequence of GspG; furthermore, PilD was required for the processing observed. Lastly, an additional band of intermediate electrophoretic mobility was detected only in the ∆*pilD*::kan background. We hypothesize this form of GspG-FLAG to be a degradation product.

### The type II secretion of the CpaA metallopeptidase is dependent on a novel protease chaperone, CpaB

**Fig 4:**
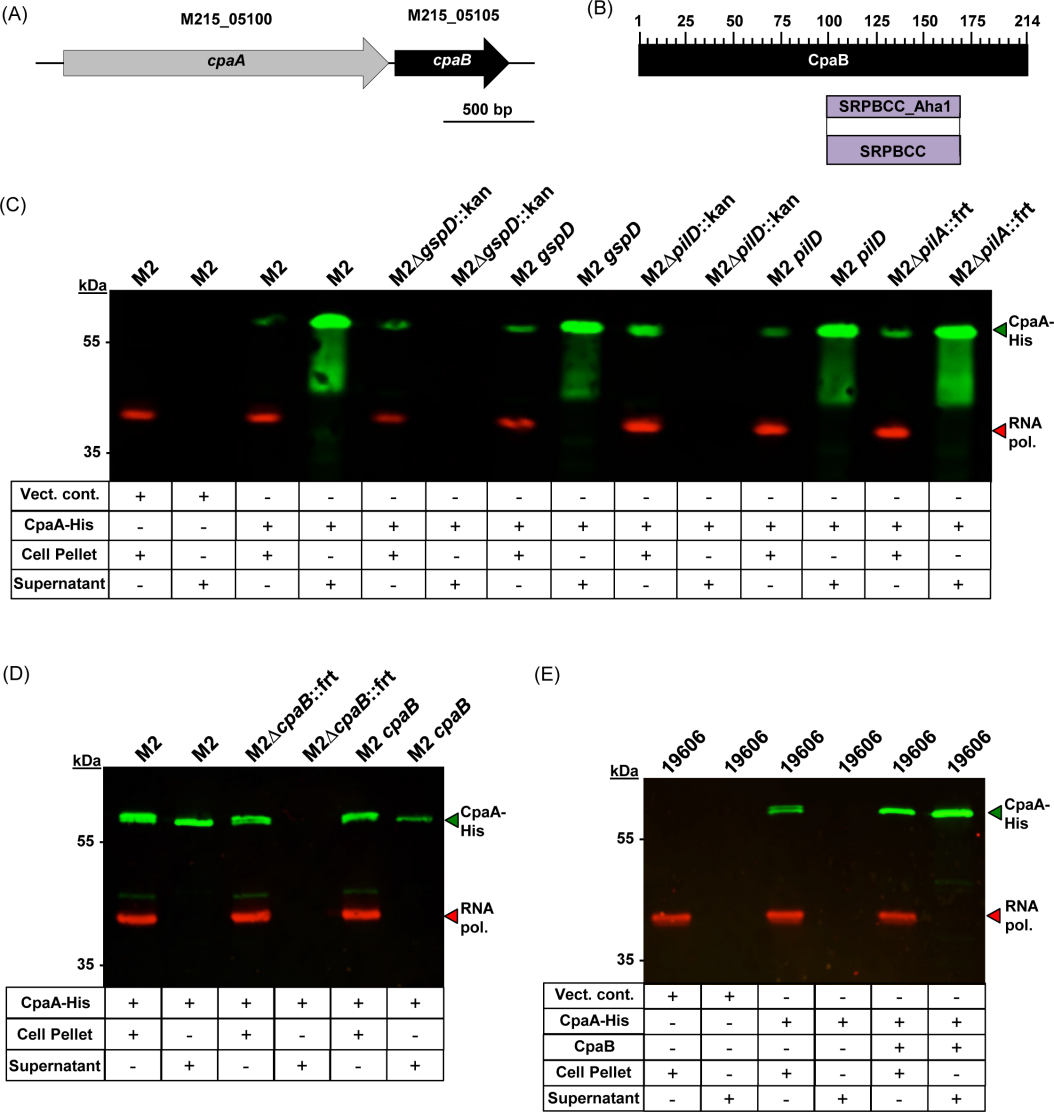
Secretion of the CpaA metallopeptidase was reliant upon both a functioning T2SS and the novel CpaB chaperone. (A) Gene arrangement of the *cpaAB* gene cluster. (B) DELTA BLASTp analysis of the CpaB amino acid sequence identified a SRPBCC domain. (C) Western blot analysis on whole cell lysates and secreted protein fractions probing for CpaA-His. All strains and fractions were also analyzed for RNA polymerase expression, which served as a lysis control. CpaA-His expression was detected in all strains carrying pWH-*cpaA-his*; however, CpaA-His secretion was only detected in strains predicted to produce a functioning T2SS. (D) Western blot analysis on whole cell lysates and secreted protein fractions probing for CpaA-His from the parent strain, the *∆cpaB*::frt mutant, and the *cpaB* complemented strain. CpaA-His was detected in all strains; however, CpaA-His secretion was not detected in the protein fraction from the *∆cpaB*::frt mutant. (E) Western blot analysis on whole cell lysates or secreted protein fractions from *A*. *baumannii* ATCC 19606 carrying the empty vector, the pWH-*cpaA-his* vector, or the pWH-*cpaA-his-cpaB* vector. CpaA-His production was detected; however, CpaA-His secretion was only detected when the *cpaB* gene was co-expressed with *cpaA-His*.

Our 2D DIGE analysis indicated that the CpaA metallopeptidase was secreted via the T2SS; therefore, we used an immunoblotting approach to verify CpaA secretion was type II dependent. We cloned the *cpaA* gene with its predicted native promoter into the *Acinetobacter*-*E*. *coli* shuttle vector pWH1266 [54], containing a hexa-histidine tag onto the carboxy terminus of *cpaA*. Hexa-histidine tagged CpaA was expressed *in trans* in multiple genetic backgrounds to probe for expression and secretion. CpaA-His expression was detected in all strains tested, however, it was only detected in the secreted fractions from strains predicted to have a fully functioning T2SS (Fig 4C). Specifically, neither the ∆*gspD*::kan mutant nor the *∆pilD*::kan mutant secreted CpaA-His, indicating the dependency of the T2SS for active export of CpaA. As expected secretion was independent of the type IV pilus as the ∆*pilA*::frt mutant displayed active secretion of CpaA-His.

Immediately downstream of *cpaA* is the M215_05105 open reading frame, which when analyzed by BLASTp did not identify any known functional domains. However, when the M215_05105 ORF was analyzed by Domain Enhanced Lookup Time Accelerated (DELTA) BLASTp, which has higher sensitivity than BLASTp [55], the M215_05105 ORF was found to contain a domain from the SRPBCC superfamily (Fig 4A and 4B). Proteins carrying a domain from the SRPBCC superfamily are predicted to contain a deep hydrophobic ligand-binding pocket and have chaperone-like activity [56]. We thus hypothesized that the M215_05105 gene product, designated CpaB due to its proximity to CpaA, was a CpaA-specific chaperone. To test our hypothesis, we deleted the *cpaB* gene and probed for CpaA-His expression and secretion. As shown in Fig 4D, CpaA-His expression was detected in the *∆cpaB*::frt mutant; however, CpaA-His was not secreted, indicating that CpaB was required for CpaA secretion. Importantly, we were able to reintroduce the *cpaB* allele and restore the active secretion of CpaA-His.

To further demonstrate the dependency of CpaA secretion on CpaB, we heterologously expressed *cpaA*-*his* alone or in tandem with *cpaB* in *A*. *baumannii* ATCC 19606, which does not encode for orthologs of either the CpaA metallopeptidase or the CpaB chaperone, yet is predicted to produce a functional T2SS. As shown in Fig 4E, CpaA-His was expressed but not secreted by 19606 cells when the pWH-*cpaA*-*his* plasmid was introduced, however, when both *cpaA-his* and *cpaB* were co-expressed, CpaA-His was secreted, indicating that CpaA secretion is not only dependent on a functional T2SS, but also on the chaperone activity of CpaB.

### The LipA lipase is exported by the type II secretion system, is lipolytic towards neutral triglycerides, and is dependent on the LipB chaperone for secretion

**Fig 5:**
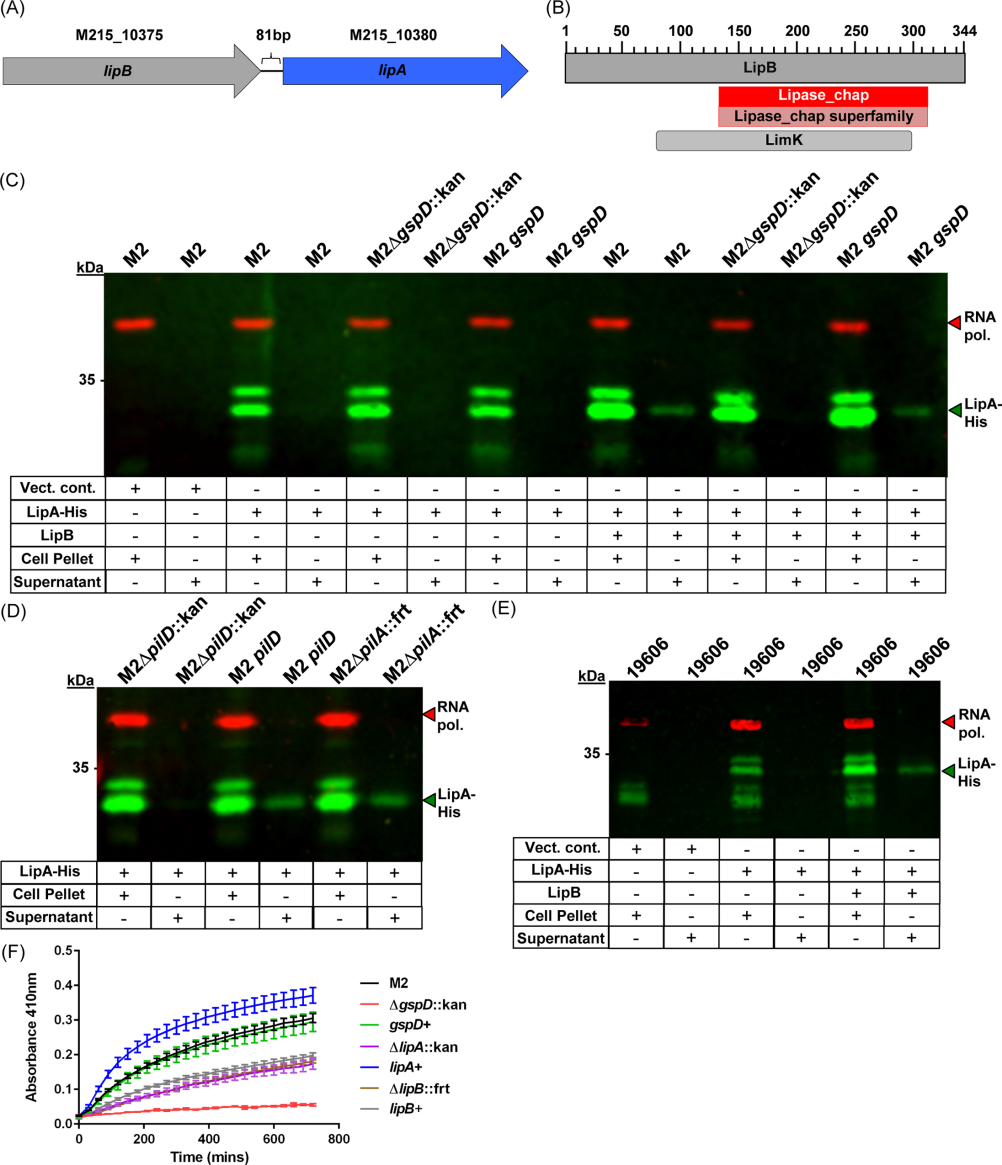
Secretion of a LipA lipase ortholog was dependent on the type II secretion system and the LipB chaperone. (A) Gene arrangement of the *lipBA* gene cluster. (B) BLASTp analysis of the LipB amino acid sequence identified multiple domains associated with steric chaperone activity including the LimK domain and a lipase chaperone superfamiliy domain. (C) Western blot analysis on whole cell lysates and secreted protein fractions probing for LipA-His. All strains and fractions were also analyzed for RNA polymerase expression, which served as a lysis control. LipA-His expression was detected in all strains carrying pWH-*lipA-his* as well as pWH-*lipB-lipA-his*; however, LipA-His secretion was only detected in strains co-expressing the chaperone LipB as well as a predicted functioning T2SS. (D) Secretion of LipA-His was independent of the type IV pilus, as indicated by active secretion in the *∆pilA*::frt mutant. (E) Western blot analysis on whole cell lysates or secreted protein fractions from *A*. *baumannii* ATCC 19606 carrying the empty vector, the pWH-*lipA-his* vector, or the pWH-*lipB-lipA-his* vector. LipA-His expression was detected; however, LipA-His secretion was only detected when the *lipB* gene was co-expressed with *lipA-His*. (F) Lipolytic activity of purified culture supernatants from the indicated strains as determined by a modified *p*-NPP assay. Increases in the A_410_ indicate lipolytic activity. A_410_ measurements were recorded every 30 minutes for 12 hours. Three biological replicates with three technical replicates were used for analysis.

The M215_10380 ORF, encoding for a LipA ortholog, was also identified in our 2D-DIGE analysis as a type II effector. It has been previously demonstrated in *A*. *baylyi* and *Pseudomonas* that secretion and over-expression of LipA orthologs are dependent on a LipB-like chaperone [51, 57]. In *A*. *nosocomialis* M2, a *lipB* homolog is adjacent to *lipA* (Fig 5A and 5B). When LipA-His was over-expressed from the pWH-*lipA*-*his* plasmid, we did not detect its secretion. However, when we co-expressed the upstream *lipB* gene with *lipA-his*, LipA-His was expressed and secreted in all backgrounds predicted to have a functional T2SS (Fig 5C). LipA-His was neither detected in the secreted fraction from the *∆gspD*::kan mutant nor the *∆pilD*::kan mutant. Secretion was also independent of the type IV pilus fiber itself (Fig 5D). We also confirmed that LipA was secreted in a LipB chaperone-dependent manner by *A*. *baumannii* ATCC 19606 (Fig 5E).

To confirm that LipA is in fact a lipase, we purified culture supernatants from multiple genetic backgrounds and probed for lipolytic activity as determined by a modified para-nitrophenol palmitate (*p-*NPP) assay [58]. As seen in Fig 5F, culture supernatants from the wild type M2 exhibited lipolytic activity as demonstrated by an increase in the absorbance at 410nm (A_410_) over a 12-hour time period. Culture supernatants from the ∆*gspD*::kan mutant displayed only minimal increases in the A_410_ indicating almost a complete lack of lipase activity. Importantly, the complemented *gspD* strain displayed very similar increases in the A_410_ when compared to the wild type, indicating that the lipase activity in culture supernatants was mainly dependent on the T2SS. Culture supernatants from the *lipA*::kan mutant exhibited an approximately 50% reduction in lipase activity; furthermore, the complemented *lipA* strain regained activity; in fact, culture supernatants from the complemented *lipA* strain displayed approximately a 30% increase in lipase activity over the wild type strain. Next we purified culture supernatants from the *lipB*::frt mutant and found that it displayed the same profile as the *lipA* mutant when measuring the A_410_; however, when we reintroduced the *lipB* gene into the *lipB*::frt mutant, we observed minimal complementation (Fig 5F).

### The M215_03235 locus encodes for a newly characterized lipase, LipH, which is also secreted in a type II dependent manner

The 2D-DIGE analysis revealed that the spot corresponding with the M215_03235 protein was associated with an 8.1 fold change when compared to the ∆*gspD*::kan mutant. The M215_03235 gene encodes for a protein containing multiple predicted domains including a LIP domain (pfam03583), a DAP2 domain (COG1506), and two AB hydrolase_5 domains (pfam 12695). Given that all of these domains are associated with predicted lipase/esterase activity, we have designated M215_03235 as *lipH* in order to avoid confusion with previously characterized lipases.

**Fig 6:**
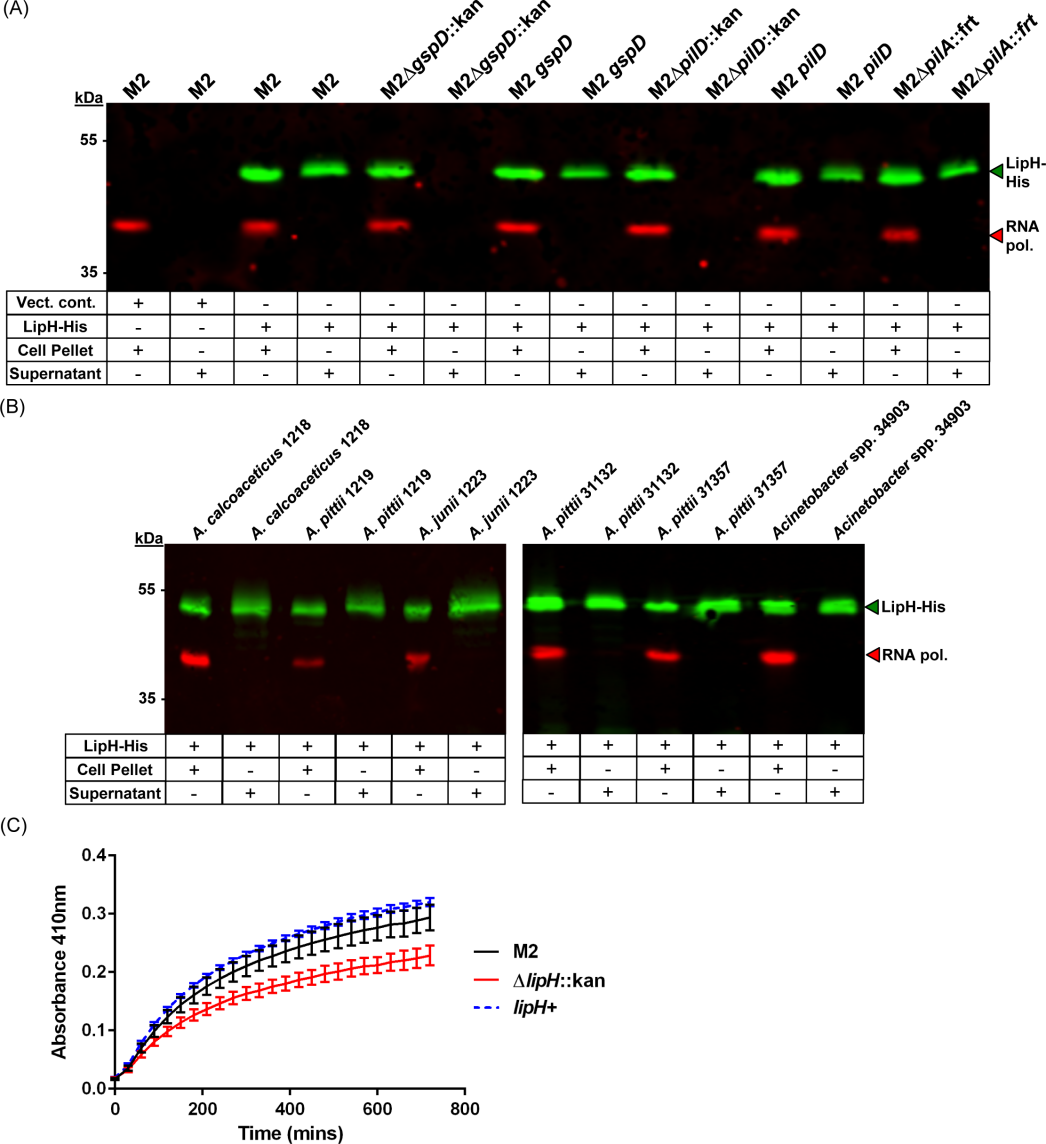
The newly characterized LipH is secreted by the T2SS and displays lipase activity. (A) Western blot analysis on whole cell lysates and secreted protein fractions probing for LipH-His. All strains and fractions were also analyzed for RNA polymerase expression, which served as a lysis control. LipH-His expression was detected in all strains carrying the pWH-*lipH-his*; however, LipH-His secretion was only detected in strains predicted to produce a functioning T2SS. (B) Western blot analysis on whole cell lysates and secreted protein fractions from a range of *Acinetobacter* clinical isolates heterologously expressing LipH-His from the pWH-*lipH-his* plasmid. LipH-His expression and secretion was detected in all clinical isolates tested. (C) Lipase activity of concentrated culture supernatants from the parent strain, the ∆*lipH*::kan mutant, and the *lipH* complemented strain. Increases in the A_410_ indicate lipase activity. A_410_ measurements were recorded every 30 minutes for 12 hours. Three biological replicates with three technical replicates were used for analysis.

To confirm that LipH was secreted in a T2SS-dependent manner, we utilized a similar approach as described above where we cloned and tagged LipH into pWH1266 with a carboxy-terminal his tag. We then introduced this construct into multiple strains and probed from LipH-His expression and secretion. As seen in Fig 6A, LipH-His was detected in whole cell lysates of all strains tested; however, LipH-His was found to only be secreted in strains predicted to express a functional T2SS. We further assessed the ability of a panel of clinical isolates to secrete LipH-His. As shown in Fig 6B, LipH-His expression and secretion was detected in all clinical isolates tested.

Because alpha/beta hydrolase domains, such as the one present in LipH, are commonly found in lipases, we verified that LipH has lipolytic activity. We constructed a ∆*lipH*::kan mutant as well as a *lipH* complemented strain and subjected these strains to the *p*-NPP assay utilized above for LipA. As seen in Fig 6C, the ∆*lipH*::kan mutant displayed an increase in the A_410_, indicating lipolytic activity; however, the increase was substantially lower than both the parent strain as well as the *lipH* complemented strain indicating that the LipH protein is a lipase.

### The *Acinetobacter* T2SS is required for optimal virulence in the *Galleria mellonella* model of infection

**Fig 7:**
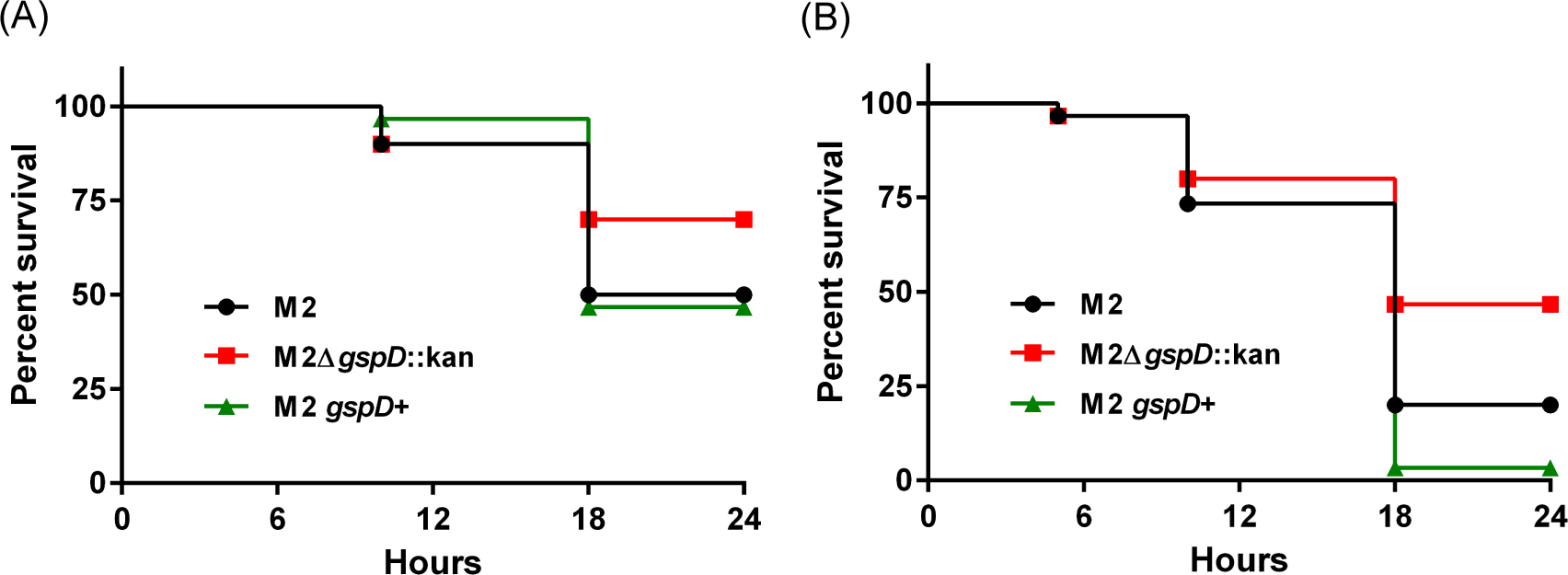
The T2SS of *A*. *nosocomialis* strain M2 is required for optimal virulence in the *G*. *mellonella* infection model. (A) Groups of *G*. *mellonella* larvae were injected with 10μL of either the parent strain, the *∆gspD*::kan mutant, or the complemented *gspD*::kan strain at an inoculum previously determined to be the equivalent of the LD_50_ for the parent strain. Larvae were checked for viability as determined by melanin accumulation and motility. (B) Groups of *G*. *mellonella* were injected with 10μL of either the parent strain, the *∆gspD*::kan mutant, or the complemented *gspD*::kan strain at an inoculum previously determined to be the LD_50_ for the *∆gspD*::kan mutant. Larvae were checked for viability as determined by melanin accumulation and motility. Survival curves were determined to be statistically significant using the Mantel-Cox test (P = 0.0147).

The greater wax moth, *Galleria mellonella*, has been routinely used to assess the virulence of *Acinetobacter* [59]. Furthermore, strains with attenuated virulence in the *G*. *mellonella* model have also been shown to have attenuated virulence in murine models of infection [60]. In order to assess the role of the *Acinetobacter* T2SS in the *G*. *mellonella* model, we first determined the LD_50_ for the wild type *A*. *nosocomialis* strain M2. Groups of ten larvae were each injected with 10μL of either approximately 10^5^, 10^6^, or 10^7^ total CFU of strain M2, incubated at 37°C for 24 hours, and checked for viability as determined by accumulation of melanin and loss of movement. From these studies, the LD_50_ was determined to be approximately 3X10^6^ CFU and was selected as the inoculation dose for subsequent infections (S1 Fig). The wild type M2, *gspD*::kan mutant, and the *gspD* complemented strain were individually injected into cohorts of *G*. *mellonella* at the specified dose, incubated at 37°C for 24 hours and checked for viability. As expected, 50% of the larvae injected with either the wild type M2 or complemented *gspD* strain succumbed to the infection (Fig 7A); however, only 30% of the larvae injected with the M2*∆gspD*::kan mutant died after 24 hours. To further demonstrate that the *Acinetobacter* T2SS contributes to the virulence of *Acinetobacter* in the *G*. *mellonella* model, we injected cohorts of larvae with the pre-determined LD_50_ for the M2*∆gspD*::kan mutant of 10^7^ CFU (S2 Fig). As seen in Fig 7B, 50% of the larvae injected with M2*∆gspD*::kan mutant died at the specific dose; however, 80% of the larvae injected with the wild type M2 died as a result of the infection after 24 hours. Interestingly, almost all of the larvae (~97%) injected with the complemented *gspD* strain died after 24 hours.

### The T2SS is required for optimal colonization of both the lungs and spleen in a murine pulmonary infection model

**Fig 8:**
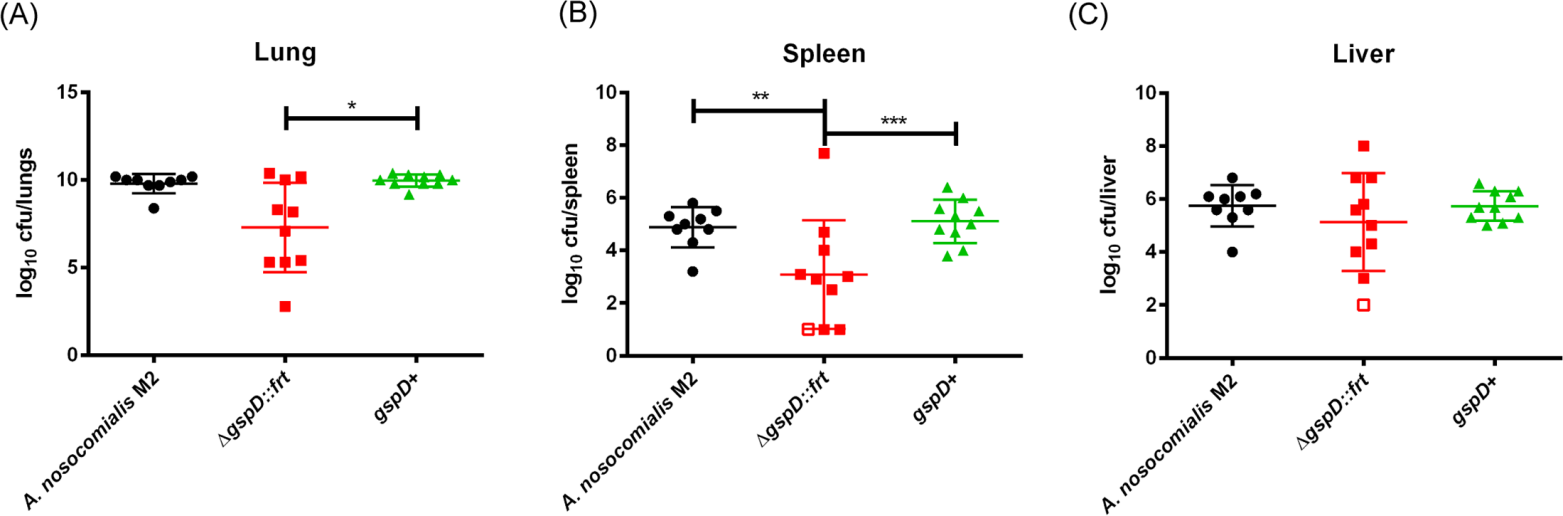
*A*. *nosocomialis* strain M2 required its T2SS for optimal colonization of both the lungs and spleen. Mice were intranasally inoculated with 1X10^9^ CFU of either the wild type, *∆gspD*::frt mutant, or the *gspD* complemented strain. After 36h post-infection, mice were sacrificed, organs harvested, and CFUs enumerated from homogenized tissue. (A) Total CFUs from the lung demonstrated a statistically significant difference between the complemented *gspD* strain and the *∆gspD*::frt mutant (Kruskal-Wallis non-parametric with Dunn’s multiple comparison test, ANOVA, *p = 0.0467). (B) Total CFUs from the spleen demonstrated statistically significance comparisons for both comparisons of either the wild type and the *∆gspD*::frt mutant as well as the complemented *gspD* strain and the *∆gspD*::frt mutant (One way ANOVA, **p = 0.0230, ***p = 0.0078). Open boxes indicate CFUs that were below the limit of detection. (C) Total CFUs form the liver did not demonstrate any statistically significant differences. Open boxes indicate CFUs that were below the limit of detection

*Acinetobacter* infections most frequently manifest as pneumonias, specifically, within the mechanically ventilated patient population [61]. The murine acute pulmonary infection model has therefore been developed to model an active *Acinetobacter* pneumonia clinical presentation. In order to determine a role of the T2SS in *Acinetobacter* virulence, we first constructed a strain of *A*. *nosocomialis* with an unmarked, in-frame deletion of *gspD*, which encodes for the predicted outer-membrane secretin. Prior to infection studies, we verified that the newly generated M2∆*gspD*::frt mutant was in fact impaired in secretion of type II effector proteins (S3 Fig). Using our previously described murine infection model [62], we performed infection experiments with either the wild type *A*. *nosocomialis* strain M2, the unmarked, isogenic M2*∆gspD*::frt mutant, or its respective *gspD* complemented strain. Mice were intranasally inoculated with 1X10^9^ CFU, as we previously determined that inoculating mice with this dose of wild type bacteria resulted in full murine viability, yet, resulted in significant organ-specific bacterial burden (S4 Fig). Groups of mice were individually administered an intransal inoculation of either the wild type strain, the ∆*gspD*::frt mutant, or the respective complemented *gspD* strain. Thirty-six hours post-infection, mice were sacrificed and the lungs, spleen, and livers were harvested in order to determine total bacterial burdens. As seen in Fig 8A, mice infected with either the wild type strain or the complemented *gspD* strain all had high bacterial burdens in the lungs. Furthermore, bacterial burdens displayed limited variability indicating a full level of complementation for the *gspD* complementation strain. Mice infected with the ∆*gspD*::frt mutant displayed significantly lower bacterial burdens in the lung when compared to either the wild type or complemented *gspD* strain. A similar trend was also observed for bacterial burdens in the spleen, where, mice infected with either the wild type or the complemented *gspD* strain had significantly higher bacterial burdens (Fig 8B). We also enumerated bacterial colony forming units from the livers of infected mice and did not observe any significant differences between the cohorts (Fig 8C).

### LipB and CpaB belong to a distinct class of membrane-bound T2SS chaperones found in Gram-negative bacteria

We have shown that two of the three secreted type II effectors identified in *A*. *nosocomialis* strain M2 require specific chaperones for secretion. To date only the lipase-specific foldases (Lifs) have been characterized as chaperones for type II effectors [51, 63–65]. Indeed, a complex of the *B*. *glumae* LipA/Lif has been crystallized [66]. The Lifs are unique steric chaperones, which have an N-terminal membrane-anchor and a C-terminal domain that facilitates proper folding of their cognate lipase upon entry into the periplasm [67]. Furthermore, the first characterization of a chaperone participating in the secretion of a type II secreted protein from *Acinetobacter* was described in 1995. These authors demonstrated that a lipase specific chaperone, designated LipB, was required for secretion of the LipA lipase. They found that the C-terminal domain of the LipB chaperone was located outside of the cytoplasm. Lastly, in contrast to what had been previously found in *Pseudomonas* strains, the authors found that *lipB* was actually encoded upstream of *lipA* [51, 52]. We have expanded upon this paradigm with the identification of a novel protease/chaperone pair (CpaA/B). Furthermore, we hypothesized this phenomenon to be more widespread. In order to identify putative chaperones of type II secreted proteins, we first searched for open reading frames (ORFs) encoded adjacently to known type II effectors that were predicted to be part of the same operon. We then narrowed our search to ORFs that encode for proteins with a predicted N-terminal transmembrane domain as this feature is shared both by the Lifs and the newly characterized CpaB chaperone. As found in Table 1, we were able to identify several putative chaperones of type II effectors in diverse Gram-negative bacteria such as *V*. *cholerae*, *P*. *aeruginosa*, and *B*. *pseudomallei*, which suggests that CpaB, LipB, and Lifs belong to a family of membrane-bound chaperones involved in T2SS secretion.

**Table 1:** Known and putative type II chaperones. Open reading frames of unknown function within the same operon as known type II effectors were analyzed for N-terminal transmembrane helices. Transmembrane domains were predicted using the TMHMM Server v. 2.0. *The *lipB* annotation in *P*. *aeruginosa* PAO1 is truncated, excluding the N-terminal sequence. Here we have included the putative full length open reading frame for analysis.

Chaperone/PutativeChaperone	Genus, species, strain	Type II effector/Function	Nucleotide Separation	Transmembrane Helix	Reference
**LipB(M215_10375)**	*Acinetobacter nosocomialis* M2	LipA (M215_10380)/Lipase	81bp	7–29	This study
**CpaB (M215_05105)**	*Acinetobacter nosocomialis* M2	CpaA (M215_05100)/Protease	22bp	7–26	This study
Hypothetical (M215_03240)	*Acinetobacter nosocomialis* M2	LipH (M215_03235)/Lipase	62bp	26–48	This study
**LipB (ACIAD3308)**	*Acinetobacter baylyi* ADP1	LipA (ACIAD3309)/Lipase	132bp	7–26	[51, 52]
**LipB (bglu_2g7740)**	*Burkholderia glumae* BGR1	LipA (bglu_2g7730)/Lipase	-1bp	21–40	[63, 64]
Hypothetical (BURPS668_3453)	*Burkholderia pseudomallei* 668	BURPS688_3454/Peptidase S10, serine carboxypeptidase	77bp	7–26	[68]
Hypothetical (BURPS668_1220)	*Burkholderia pseudomallei* 668	BURPS688_1221/ Pectinacetylesterase	62bp	7–24	[68]
Hypothetical (BURPS668_0360)	*Burkholderia pseudomallei* 668	BURPS688_0358/Nonhemolytic phospholipase C	46bp	7–24	[68]
LipB* (PA2863)	*Pseudomonas aeruginosa* PAO1	LipA (PA2862)/Lipase	-17bp	26–44	[69–71]
LipB* (PA2863)	*Pseudomonas aeruginosa* PAO1	LipC (PA4813)/Lipase	2,185,726bp	26–44	[72]
Hypothetical (PA2872)	*Pseudomonas aeruginosa* PAO1	Mep72 (PA2783)/Protease	77bp	5–24	[73]
Hypothetical (A5E_A0255)	*Vibrio cholerae* B33	PrtV (A5E_A0254)/Protease	84bp	5–27	[74]
**LipB (VCA0222)**	*Vibrio cholera* N16961	LipA (VCA0221)/Lipase	9bp	7–26	[75]

## Discussion

*Acinetobacter* spp. have rapidly emerged as significant opportunistic pathogens afflicting healthcare facilities worldwide. Although sophisticated studies track the epidemiology of outbreaks worldwide, our collective understanding of the molecular mechanisms employed by *Acinetobacter* spp. to cause disease is in its infancy. In this work, we combined bioinformatics, proteomics, mutational analyses, and virulence assays to demonstrate that *Acinetobacter* spp. produce a functional T2SS, which is required for the secretion of multiple proteins that are required for full virulence. Importantly, this is the first *bona fide* secretion system required for virulence in a mammalian model identified in *Acinetobacter*. Notably, two of the three secreted proteins characterized in this study require dedicated chaperones for type II secretion. While this paper was under revision, an article reporting the presence of a functioning T2SS in *A*. *baumannii* ATCC 17978 was published [76]. In this work, it was found that 17978 also required a T2SS for the secretion of the LipA lipase and growth on minimal media with olive oil as the sole carbon source. It was also found that both the 17978*∆gspD* and 17978*∆lipA* mutants were less fit in a murine septicemia model when competed against the parental strain.

Typically, T2SSs secrete as many as 18–25 proteins and facilitate the delivery of major virulence factors to the extracellular environment for many important human pathogens, such as *Legionella pneumophila* and *V*. *cholerae* [40, 42]. Here, we utilized the 2D-DIGE method coupled with mutational analyses to characterize the type II secretome for *A*. *nosocomialis* strain M2. Our analysis identified over 60 spots with a 4-fold difference when comparing the wild type M2 vs. M2∆*gspD*::kan mutant; however, we concentrated our efforts on three proteins that contain domains of known functions. Other studies will be needed to determine the role of the remaining type 2 effector candidates and of individual secreted proteins in *Acinetobacter* pathobiology given the importance of this system in virulence.

The genetic architecture of T2SSs usually consists of between 12 and 15 genes, most of which appear to be organized in a single operon [35]. From a regulatory standpoint, the single operon arrangement of T2SS associated genes would seem to be the simplest to transcriptional control. However, as noted above, the T2SS associated genes from *A*. *nosocomialis* strain M2 are found in five distinct genetic loci, a genetic arrangement that resembles the type IVa pilus system [77]. Furthermore, this dispersed genetic arrangement is highly conserved across different *Acinetobacter* species, including the pathogenic species *A*. *baumannii* and the non-pathogenic species *A*. *baylyi*. Closer examination of each T2SS gene cluster does not provide any obvious insights into the regulatory mechanisms as some T2SS genes appear to be in putative operons with other genes not known to be associated with T2SSs. Outside of the genus *Acinetobacter* the same genetic architecture can also be found in bacteria from the genus *Psychrobacter*.

As demonstrated previously, the prepilin peptidase PilD was required for major pilin processing and proper functionality of T4P in *A*. *nosocomialis* strain M2 [45]. Our current data demonstrated that PilD is also required processing of the predicted major pseudopilin, GspG, and thus secretion of T2S substrates. Given the strong evolutionary relatedness between the T4P system and the T2SS, the phenomenon of sharing protein components between two functionally distinct systems does not seem impractical, nevertheless, it is uncommon. To date only *D*. *nodosus* [78], *P*. *aeruginosa* [79], *V*. *cholerae* [80], and *L*. *pneumophila* [81, 82] have been demonstrated to share a prepilin peptidase between both the T4P system and a T2SS.

Of the three type II effectors studied, only LipA has previously characterized orthologs, which were primarily described in *Pseudomonas* and also require a chaperone [83]. However, to date, none of these lipases have been connected to pathogenesis. We demonstrated that the LipA lipase was responsible for approximately half of the lipase activity observed from the secreted fraction of the wild type strain M2. As expected, LipA activity was also dependent on the LipB chaperone, as supernatants from the ∆*lipB*::frt mutant displayed nearly identical lipase activity levels as the *∆lipA*::kan mutation. However, our *lipB* complemented strain only marginally increased the lipase activity of the *∆lipB*::frt mutant, indicating that even though we constructed an in-frame, unmarked mutation in the *lipB* gene, we may still be observing polar effects on *lipA* transcription. The *lipA* gene is 81bp downstream of the *lipB* gene and therefore could potentially have its own promoter that is partially contained within the 3’ region of the *lipB* gene. We and others have observed similar cryptic promoter events during previous studies of the *pilTU* gene cluster, where an in-frame, unmarked mutation of *pilT* still had polar effects on *pilU* expression [45, 84].

Even in the absence of *lipA*, culture supernatants retained residual lipase activity as compared to the *gspD* mutant strain. As such, we found that LipH mediated lipase activity of culture supernatants as well. A BLASTp search of LipH orthologs outside of *Acinetobacter* identified similar proteins found in bacteria from the genus *Myriodes*, some of which act as opportunistic human pathogens [85], as well as bacteria from the genus *Bacillus*; however, none of those orthologs have been characterized.

Using *A*. *nosocomialis* strain M2 as our model system we demonstrated that LipH secretion was indeed dependent on a functional T2SS. We also demonstrated that T2SS is conserved and functional across *Acinetobacter* spp. via immunoblotting of epitope tagged effectors. Specifically, we showed that LipH from M2 was secreted by a panel of *Acinetobacter* clinical isolates, including, *A*. *calcoaceticus*, *A*. *baumannii*, *A*. *pittii*, *and A*. *junnii*. We also demonstrated that *A*. *baumannii* ATCC 19606 could secrete both LipA and CpaA; however, as expected the respective chaperones for each protein were required for active secretion. These data strongly suggest the presence of a functional T2SS in the majority of medically relevant *Acinetobacter spp*. This hypothesis is further supported by the fact that genes predicted to encode proteins required for the biogenesis of the T2SS are highly conserved and distributed amongst *Acinetobacter* spp.

The remaining effector characterized in our study was the CpaA metallopeptidase. CpaA was previously purified from culture supernatants [49]; however, its mechanism of secretion was not determined. It was previously shown that CpaA is involved in degradation of Factor V and fibrinogen, which would result in a decrease in clotting activity. Here, we demonstrated that CpaA was secreted in abundance in a type II dependent manner, yet, was also dependent on a novel chaperone, designated CpaB. CpaB is the first characterized T2SS chaperone devoted to the secretion of a protease. Topological modeling of the CpaB chaperone predicts a single N-terminal transmembrane domain with the majority of the protein exposed to the periplasm [86, 87]. The periplasmic exposed C-terminal domain of CpaB was predicted by DELTA BLASTp to contain a domain from the SRPBCC superfamily present in the co-chaperone eukaryotic protein Aha1, the activator of Hsp90 complex [56]. The SRPBCC domains are predicted to have deep hydrophobic ligand binding pockets. A BLASTp search of CpaB orthologs outside of *Acinetobacter* only identified two weak orthologs from *Lysobacter antibioticus*; however, a DELTA BLASTp search for CpaB orthologs outside of *Acinetobacter* primarily identified Aha1 as the closet ortholog, suggesting a possible eukaryotic ancestry. Currently, we hypothesize that the CpaA metallopeptidase is trafficked through the Sec system, as is the case for most type II secreted substrates. There, CpaA can interact with CpaB as CpaB is predicted to contain a single transmembrane domain with the majority of the protein exposed to the periplasmic space. Upon entry into the periplasmic space of CpaA from the Sec system, CpaB could facilitate proper folding of CpaA due to the requirement of type II secretion systems for competently folded proteins for active secretion.

The potential role of the CpaA metallopeptidase in *Acinetobacter* pathogenesis and evolution is quite intriguing. Firstly, the type strains *A*. *baumannii* ATCC 17978 and 19606, two of the more primitive *Acinetobacter* spp. used as model organisms do not contain orthologs of the CpaAB system, indicating a horizontal acquisition event within the last 70 years. Analysis of the GC content of the *cpaAB* locus and the surrounding DNA support this hypothesis. It is tempting to speculate, that given the predicted recent acquisition of the CpaAB protease/chaperone system and the role of the T2SS in *Acinetobacter* virulence, CpaA may be one of the major virulence factors of some pathogenic *Acinetobacter* spp. Future work will be aimed at deciphering the role of CpaA in the virulence assays utilized within this study.

As mentioned above, LipB and CpaB act as specific chaperones for LipA and CpaA respectively. Some effectors secreted via a type III secretion system (T3SS) also require specific chaperones that have collectively been named “T3SS chaperones” [88]. T3SS chaperones do not present sequence similarity, but they are easily identified because they are encoded next to their cognate effector and most of them contain similar molecular weight and isoelectric points. Similarly, we define a “T2SS chaperone” as a protein encoded adjacently and co-regulated with a type II effector, that contains both an N-terminal transmembrane domain, and an exposed C-terminal region to the periplasm, and that is required for secretion of the cognate effectors. We identified “type II chaperones” in multiple Gram-negative species. Interestingly, LipB from *Pseudomonas aeruginosa* is a previously characterized chaperone that serves two T2SS effectors, LipA which is encoded next to LipB as well as LipC, which is encoded more than 2 Mb away [72]. This indicates that the T2SS chaperones family may be more widespread than we propose here.

We determined that the *Acinetobacter* T2SS was required for virulence. We first determined that the mutants unable to produce a functioning T2SS were attenuated in the *G*. *mellonella* infection model. Given the high level of concordance between mutants attenuated in the *G*. *mellonella* model and mammalian models, we hypothesized a more relevant *in vivo* role for the *Acinetobacter* T2SS. We thus choose to investigate the role of T2S in a murine pulmonary infection model. Specifically, we observed high CFUs for the wild-type strain in the lungs after 36h infection period and also observed dissemination to both the liver and spleen. Using an unmarked, in-frame deletion of *gspD* strain and its respective complemented strain, we were able to demonstrate that the T2SS was indeed required for optimal colonization of both the lungs and spleen, but not the liver. Remarkably, we observed almost a two log decrease in CFUs in the lungs and spleen of mice infected with the *gspD* mutant strain when compared to either the wild type or the complemented strain. Many studies focusing on *Acinetobacter* pathobiology have utilized a similar murine pneumonia model of infection and also observed differences of around 2 logs; however, these mutants had defects in two-component regulatory systems, metabolism, and/or stress responses, all of which could have more pronounced global effects on *Acinetobacter* biology that mediate defects in colonization [32, 89].

Herein, we have provided evidence of both a functional T2SS in many *Acinetobacter* spp. as well as demonstrated its importance in *Acinetobacter* pathogenicity. However, the exact role for each T2S effector proteins in *Acinetobacter* pathogenicity has yet to be determined. As such we plan to next probe the role of specific effectors in mediating the colonization phenotypes observed, with an emphasis on the most highly secreted protein, the CpaA metallopeptidase. Furthermore, our study highlights the use of other clinically relevant members of the genus *Acinetobacter* outside of *A*. *baumannii* in order to gain insights into the pathogenesis of clinically relevant *Acb* members. Although type strains like *A*. *baumannii* ATCC 17978 and 19606 have served well as model strains for *Acinetobacter* pathogenicity, their relative old age makes them less representative of current epidemic strains, which contain more antibiotic resistance cassettes and possibly novel virulence attributes.

## Materials and Methods

### Strains, plasmids, and growth conditions

Bacterial strains and plasmids utilized within this study can be located in the S1 Table. All bacterial strains were grown on L-agar at 37°C. Antibiotic selection for *E*. *coli* strains was used at the following concentrations: 100μg ampicillin/mL, 5μg tetracycline/mL, and 20μg kanamycin/mL. Antibiotic selection for *Acinetobacter* strains was used at the following concentrations: 200μg ampicillin/mL, 5μg tetracycline/mL, 20μg kanamycin/mL, 12.5μg chloramphenicol/mL. Sucrose was used at a final concentration of 10% for counter selecting *Acinetobacter* strains that lost the *sacB* cassette.

### Generation of bacterial mutants and complemented mutants

All marked and unmarked mutants were generated using the previously published methodologies found in [22, 45] using the In-Fusion HD EcoDry cloning kit. The In-Fusion HD EcoDry cloning kit was used to generate the interrupted gene constructs as described in the supplemental material of [22] and introduced into strain M2 via natural transformation as described in [45]. For strains containing the kan-*sacB* cassette, a tri-parental mating strategy was used to transiently introduce the pFLP2 plasmid as described in [23], in order to replace the cassette with an frt scar. Strains designated with the “::frt” nomenclature contain a frt scar in place of the target gene. Each mutation was complemented using the mTn*7* described in [22]. A complete list of primers for mutational analyses can be found in S2 Table.

### Bioinformatic analysis

The Basic Local Alignment Search Tool (BLAST) tool was utilized in order to identify known gene homologs of type II secretion system related genes in *Acinetobacter*.

### One dimensional SDS-PAGE analysis of secreted proteins

Fifty milliliter cultures of each strain was grown for 18 h in M9 salts supplemented with 1% casamino acids and 1% glucose with 180 rpm. The secreted proteins were separated from the whole cells by centrifugation at 4000rpm for 10 mins. The supernatants were then further purified by filtration through 0.22 micron filters. The secreted proteins were then concentrated to ~100μL using Amicon Ultra Centrifugal Filter units with a 10kDa cutoff. Laemmli buffer with β-mercaptoethanol was added to each fraction and the samples were heated to 100°C by boiling in water for 10 mins. Twenty microliters of each sample was then separated by SDS-PAGE in a 4–20% gradient gel and subsequently silver stained.

### 2D-DIGE analyses

Secreted proteins used for the 2D-DIGE analysis were prepared as described in the above section discussing 1D SDS-PAGE analysis of secreted proteins for both the wild type *A*. *nosocomialis* strain M2 and its isogenic *gspD*::kan mutant. A detailed protocol for the 2D-DIGE analysis can be located in S1 Appendix. All 2D-DIGE experiments were performed by the Campus Chemical Instrument Center Mass Spectrometry and Proteomics Facility at The Ohio State University.

### Generation of pWH1266 carrying effectors and effector/chaperone pairs

In order to validate the 2D-DIGE analysis identifying the putative type II secreted proteins of strain M2, selected effectors and effector/chaperone pairs were cloned into the *Acinetobacter-E*. *coli* shuttle vector pWH1266. Briefly, *lipA*, *cpaA*, *lipH*, *lipBA*, and *cpaAB* loci were PCR amplified using the primers listed in S2 Table using strain M2 genomic DNA as template for PCRs. Each PCR product was purified, digested with PvuI-HF, and ligated into pWH1266 that was predigested with PvuI-HF and treated with phosphatase. The ligations were transformed into *E*. *coli* TOP10 cells and transformants were selected for on L-agar supplemented with tetracycline. Transformants were sub-cultured and each plasmid was purified and verified by sequencing. The carboxy-terminal His tag was added to *lipA*, *lipH*, and *cpaA* with a second PCR, where the respective forward primer included a 5’ overhang encoding for the His-tag using with the primers listed in S2 Table. The PCR products were purified, DpnI treated, and self-ligated. The ligations were transformed into TOP10 cells and transformants were selected on L-agar supplemented with tetracycline. Transformants were sub-cultured and the plasmids were purified and verified by sequencing. Vectors expressing the His-tagged constructs were electroporated into electrocompetent *Acinetobacter* spp. and transformants were selected for on L-agar supplemented with tetracycline.

### Generation of pWH-*gspG*-FLAG

To test for PilD-dependent processing of GspG, the *gspFG* locus including the predicted native promoter was PCR amplified using the primers listed in S2 Table. The PCR product was purified, digested with PvuI-HF, and ligated into pWH1266 that was predigested with PvuI-HF and treated with phosphatase. The ligations were transformed into TOP10 cells and transformants were selected on L-agar supplemented with tetracycline. Transformants were sub-cultured and the plasmids were purified and verified by sequencing. To remove the *gspF* gene, an inverse PCR strategy was employed to PCR out *gspF* leaving the ATG start codon and the last 21 bp in order to generate an in-frame deletion. The PCR product was purified, treated with kinase, and self-ligated. The ligations were transformed into TOP10 cells and transformants were selected on L-agar supplemented with tetracycline. Transformants were sub-cultured and the plasmids were purified and verified by sequencing. The FLAG tag was PCR amplified onto the carboxy terminus of *gspG* as described above using the primers listed in S2 Table. The PCR product was purified, treated with kinase, and self-ligated. The ligations were transformed into TOP10 cells and transformants were selected on L-agar supplemented with tetracycline. Transformants were sub-cultured and the plasmids were purified and verified by sequencing. The pWH-*gspG*-FLAG construct was then electroporated into electrocompetent *A*. *nosocomialis* strains.

### Type II secreted protein detection

Strains carrying His-tagged *lipA*, *lipH*, or *cpaA* were screened for active secretion via immunoblotting. Briefly, strains were struck and grown overnight on L-agar supplemented with tetracycline at 37°C. Bacteria were swabbed from the plate, resuspended in LB broth, and used to inoculate 10mL of LB broth to an OD_600_ of 0.05 supplemented with tetracycline. The cultures were grown to mid-log phase, normalized to an OD_600_ of 0.5, then processed for whole cell fractions and secreted fractions. Whole cell fractions were obtained by removing 1mL of the normalized mid-log cells, pelleting the cells by centrifugation, and removing the supernatant. Bacterial pellets were then resuspended in 50μL of 1X Laemmli buffer. Secreted fractions were obtained by pelleting the normalized mid-log cultures by centrifugation and carefully removing 1mL of the supernatant. Secreted proteins were precipitated by the addition of 250μL of a saturated trichloroacetic acid solution. Precipitated proteins were incubated on ice for 10–30 mins, pelleted by centrifugation, and washed twice with ice-cold acetone. Residual acetone was removed by heating the samples at 95°C. Precipitated proteins were resuspended in 100μL of 1X Laemmli buffer. Both whole cell fractions and secreted fractions were boiled in 1X Laemmli buffer for 10 mins and subsequently used for immunoblotting. Proteins were separated on a 10% SDS-PAGE gel, transferred to nitrocellulose, and probed for RNA polymerase and 6X-Histidine tagged proteins according to our previously published methodologies.

### Lipase assay

In order to determine lipolytic activity of secreted protein fractions, a modified version of the para-nitrophenol palmitate (*p*-NPP) lipase assay was performed. Secreted protein fractions were purified from select strains as described above with slight modifications. Briefly, 2.5mL of culture supernatant was clarified by centrifugation and then filtered through 0.22μM PVDF filters. The secreted proteins were buffer exchanged into 50mM Tris and were concentrated to ~250μL using Amicon Ultra Centrifugal Filter units with a 10kDa cutoff and promptly used for the lipase assay. Lipase activity was determined by measuring the absorbance at 410nm at 37°C using *p*-NPP as a substrate. The *p*-NPP solution was freshly prepared for each assay by diluting solution A (0.1g *p*-NPP in 100mL isopropanol) 1:10 with solution B (1g gum Arabic, 2g sodium deoxycholate, 5mL triton X-100, 50mM Tris-HCl pH 8 in 900mL). Seventy microliters of the *p*-NPP solution was then added to 30μL of the concentrated, clarified secreted protein fractions from a respective strain. Kinetic measurements recording the absorbance at 410nm were then performed over the designated time frame at 37°C with orbital shaking between each absorbance reading. Absorbance measurements were captured using the Synergy HTX multi-mode reader from BioTek. Each experiment was performed in triplicate with three technical replicates per sample.

### *Galleria mellonella* infection

*A*. *nosocomialis* M2, the *ΔgspD*::kan mutant and the complemented strain were grown in LB broth overnight in an orbital shaker (37°C, 200rpm). The overnight cultures were diluted to a starting OD_600_ 0.05 and grown at 37°C with 200rpm to a final OD_600_ of 0.5. 0.5ODs was pelleted by centrifugation, washed with filter sterilized PBS and resuspended at and OD of 0.5/ml, 0.158OD/mL and 0.05OD/mL in filter sterile PBS. The CFU/mL at 0.5OD/mL was determined to be 10^9^. Serial dilution of the 0.5OD/mL sample was performed. Larvae were injected with 10uL of sterile PBS, 10^6^ or 10^7^ CFU. 3 groups of 10 larvae were injected per experimental group. The larvae were scored as live/dead depending on their response to physical stimulus approximately every 5 hours. The number of bacterial cells injected into the larvae was determined by plating 10-fold serial dilutions on LB agar and performing CFU counts after overnight incubation at 37°C.

### Mouse model of pneumonia

All infection experiments were approved by the Vanderbilt University Institutional Animal Care and Use Committee. Wild-type C57BL/6 mice, obtained from Jackson Laboratories, were used for single infection experiments with either the wild type *A*. *nosocomialis* M2, the M2∆*gspD*::frt mutant, or the respective *gspD* complemented strain. Overnight cultures of each strain were sub-cultured 1/1000 into 50 mL LB broth and grown with shaking at 37°C in 250-mL flasks. Bacterial cells were harvested by centrifugation during logarithmic growth, washed twice with phosphate buffered saline (PBS), and suspended in PBS. Nine-week old male mice were inoculated intranasally with a total of 7–8 X 10^8^ cfu in 30 μL. At 36 h post-infection, mice were euthanized and CFUs were enumerated from the lungs, livers, and spleens following tissue homogenization and dilution plating to LB agar medium. The data were log transformed and analyzed for Gaussian distribution using the D’Angostino-Pearson omnibus normality test. Data sets displaying Gaussian distribution were then analyzed by One-way ANOVA with Tukey’s test for multiple comparisons. Data sets displaying non-Gaussian distribution were analyzed by Kruskal-Wallis test with Dunn’s test for multiple comparisons. All statistical analyses were performed using GraphPad Prism 6 (GraphPad Software Inc., La Jolla, CA).

### Ethics statement

Animal care and experiments were performed in accordance with the NIH “Guide for the Care and Use of the Laboratory Animals” and were reviewed and approved by the Vanderbilt University Institutional Animal Care and Use Committee (Protocol M/10/165). Mice were anesthetized with 2,2,2,-tribromoethanol prior to intranasal inoculation. Mice were euthanized by carbon dioxide.

## Supporting Information

S1 Appendix 2D-DIGE methodology and detailed results. (DOCX)

S1 Table Plasmid and strain list. (DOCX)

S2 Table Primers used in this study. (XLSX)

S1 Fig LD_50_ determination for *G*. *mellonella* larvae infected with *A*. *nosocomialis* strain M2. Groups of 10 *G*. *mellonella* were injected with 10μL of *A*. *nosocomialis* strain M2 at 3X10^5^, 3X10^6^, or 3X10^7^ CFUs. Eighteen hours after injection larvae were checked for viability as determined by melanin accumulation and motility.
(TIF)

S2 Fig LD_50_ determination for *G*. *mellonella* larvae infected with the M2*∆gspD*::kan mutant. Groups of 10 *G*. *mellonella* were injected with 10μL M2***∆*** *gspD*::kan mutant at 3X10^6^, 1X10^7^, or 3X10^7^ CFUs. Eighteen hours after injection larvae were checked for viability as determined by melanin accumulation and motility.
(TIF)

S3 Fig Type II secretion is impaired in the M2*∆gspD*::frt mutant. Western blot analysis on whole cell lysates and secreted protein fractions probing for LipH-His. All strains and fractions were also analyzed for RNA polymerase expression, which served as a lysis control. LipH-His expression was detected in all strains carrying the pWH-*lipH-his*; however, LipH-His secretion was only detected in the parental M2 strain and the complemented *gspD*::frt strain, but not the ***∆*** *gspD*::frt strain.
(TIF)

S4 Fig Dose determination of *A*. *nosocomialis* strain M2 for the murine pulmonary infection experiments. Four groups of three mice were intranasally inoculated with either 3X10^7^, 3X10^8^, 1X10^9^, or 3X10^9^ CFU of *A*. *nosocomialis* strain M2. Thirty six hours post infection surviving mice were sacrificed and organs were harvested for CFU enumeration. A single mouse from the 1X10^9^ CFU dose group had to be removed post anesthesia and was excluded from this analysis.
(PDF)
