# Supplementary material for: Medically Relevant Acinetobacter Species Require a Type II Secretion System and Specific Membrane-Associated Chaperones for the Export of Multiple Substrates and Full Virulence
Source: PLoS Pathog. 2016 Jan 14;12(1):e1005391. doi: 10.1371/journal.ppat.1005391 (PMC4713064; doi:10.1371/journal.ppat.1005391)
Supplement: S1 Appendix — (DOCX) [file ppat.1005391.s001.docx]

**S1 Appendix. 2D-DIGE experimental approach and results.**

**Materials and Methods:**

**Sample Prep:**

Samples were received in lysis B and stored immediately at -80°C until further processing. 2D cleanup was performed on 100 μg of sample according to the manufacturer’s instructions (GE, 80-6484-51). Finally, samples were suspended in 100 µL of lysis buffer (30M Tris pH 8.5, 7M Urea, 2M Thiourea, 4% CHAPS) and quantitated by Bradford assay using BSA as a standard.

**Sample Labeling:**

25 µg of each sample was labeled with the appropriate CyDye (either Cy3 or Cy5, see Table 1). Labeling reactions were performed on ice, light-protected to avoid dye deterioration and were carried out with a ratio of 400 pmol Dye/50 µg sample. 12.5 µg of each sample (100 µg total) was pooled to create an internal standard (IS) and this pooled sample was labeled with Cy2 (800 pmol). After 30 minutes, lysine was added to quench the reaction. Additionally, 52.5 µg of each sample (420 µg total) was left unlabeled and combined to run 2 preparative gels for spot-picking proteins of interest.

**Table 1: Experimental Design for 30536**

| **Gel ID** | **Cy3** | **Cy5** | **Cy2** |
| --- | --- | --- | --- |
| 1. 46640 | M2-1 | M2Δ*gspD*::kan-3 | 25 μg internal standard |
| 1. 46641 | M2Δ*gspD*::kan-2 | M2-2 | 25 μg internal standard |
| 1. 46642 | M2-3 | M2Δ*gspD*::kan-5 | 25 μg internal standard |
| 1. 46643 | M2Δ*gspD*::kan-4 | M2-5 | 25 μg internal standard |
| 1. 46644 | Preparative gel for spot picking proteins of interest | | |
| 1. 46645 | Preparative gel for spot picking proteins of interest | | |

**2D Electrophoresis: 1^st^ Dimension**

Labeled samples were combined as shown in Table 1, vortexed, and diluted with rehydration buffer (7M Urea, 2M thiourea, 2% CHAPS, 1% pH 3-10 IPG buffer (GE Healthcare), 50mM DTT, 1% saturated bromophenol blue solution) to a final volume of 450 µL. Samples were centrifuged for 20 minutes at 4 degrees Celsius/16,000 RCF, and each sample was then used to rehydrate a 24cm immobiline pH 3-10 IEF strip (GE Healthcare) under mineral oil overnight. The IEF strips were then focused on an IPGphor II (GE Healthcare) in a ceramic manifold at 20 degrees Celsius. Focus was set at 75 µA per strip maximum, focused at 500V for 1 hour, a gradient to 1000V over 1 hour, a gradient to 10,000V over 3 hours, and a hold at 10,000V for 3.25 hours. IEF strips were placed in plastic, wrapped in aluminum foil and stored at -80 degrees Celsius until further processing.

**2D Electrophoresis: 2^nd^ Dimension**

Strips were equilibrated at room temperature in 5ml of equilibration buffer A (50mM Tris pH 8.8, 6M urea, 30% glycerol, 2% SDS, 0.5% DTT w/v) for 15 minutes, followed by 5ml of equilibration buffer B (50mM Tris pH 8.8, 6M urea, 30% glycerol, 2% SDS, 4.5% iodoacetamide w/v). The reduced and alkylated strips were rinsed briefly in 1x SDS-PAGE running buffer (50mM Tris, 384 mM glycine, 0.2% SDS) and placed in 20x24cm 12% SDS-PAGE gels. Strips were sealed in place using 0.5% agarose in 2x SDS-PAGE running buffer containing 1% saturated Bromophenol blue solution. Gels were run in a Dalt 12 electrophoresis system (GE Healthcare) at 2 watts per gel for 45 minutes, followed by 15 watts per gel until the dye front reached the bottom (~4 hours). Gels (still in glass plates) were rinsed with water and immediately scanned in a Typhoon 9400 variable mode scanner (GE Healthcare) using the appropriate settings for CyDye fluorophors, scanning for cy2, then cy3, and then cy5 labels. Gels were prescanned at 1000 micron resolution to optimize PMT for each dye and then scanned at 100 micron resolution for final images. Gel images were cropped for analysis and saved. Preparative gels (for spot picking and identification) were fixed and stained with Lava purple general protein stain (Gel Company) according to standard protocols.

**In-Gel Digestion**

The Ettan Spot Handling Workstation was used to core protein spots of interest using a pick list and placed in a 96 well plate (User Manual from Amersham Biosciences). In gel digestion was also performed as follows; briefly, gel pieces were washed in 100 µL of 50% methanol/5% acetic acid for 15 min.  The wash step was repeated a total of 2 times. The gels spots were washed with acetonitrile for 5 minutes, then dried and resuspended with 50mM ammonium bicarbonate; this was repeated three times. Dried gels pieces were digested with sequencing grade trypsin from Promega (Madison WI). The protease was driven into the gel pieces by rehydrating them in 50 µL of sequencing grade-modified trypsin prepared at 5 µg/mL in 50 mM ammonium bicarbonate for 3 hours at 37ºC. The peptides were extracted from the polyacrylamide with 50 µl 50% acetonitrile and 5% formic acid three times. The extracted pool was dried for 30 minutes in a vacufuge and removed immediately to prevent complete drying.

**Mass Spectrometry**

*LTQ*

Capillary-liquid chromatography-nanospray tandem mass spectrometry (Nano-LC/MS/MS) was performed on a Thermo Finnigan LTQ mass spectrometer equipped with a nanospray source operated in positive ion mode.  The LC system was an UltiMate™ Plus system from LC-Packings A Dionex Co (Sunnyvale, CA) with a Famos autosampler and Switchos column switcher.  The solvent A was water containing 50mM acetic acid and the solvent B was acetonitrile.  5 microliters of each sample was first injected on to the trapping column (LC-Packings A Dionex Co, Sunnyvale, CA), and washed with 50 mM acetic acid.  The injector port was switched to inject and the peptides were eluted off of the trap onto the column.  A 5 cm 75 μm ID ProteoPep II C18 column (New Objective, Inc. Woburn, MA) packed directly in the nanospray tip was used for chromatographic separations.  Peptides were eluted directly off the column into the LTQ system using a gradient of 2-80%B over 50 minutes, with a flow rate of 300 nl/min.  The total run time was 60 minutes.  The MS/MS was acquired according to standard conditions established in the lab. Briefly, a nanospray source operated with a spray voltage of 3 KV and a capillary temperature of 200P^oP^C is used. The scan sequence of the mass spectrometer was based on the TopTen™ method; the analysis was programmed for a full scan recorded between 350 – 2000 Da, and a MS/MS scan to generate product ion spectra to determine amino acid sequence in consecutive instrument scans of the ten most abundant peaks in the spectrum. The CID fragmentation energy was set to 35%. Dynamic exclusion was enabled with a repeat count of 30 s, exclusion duration of 350 s and a low mass width of 0.5 and high mass width of 1.50 Da.

*Bioinformatics*

Sequence information from the MS/MS data was processed by converting the raw dta files into mascot generic files (.mgf) using MS Convert (ProteoWizard). The resulting mgf files were searched against other acinetobacter in the NCBI database (3,487 sequences) using Mascot Daemon by Matrix Science version 2.2.1 (Boston, MA). The mass accuracy of the precursor ions were set to 1.8 Da given that the data was acquired on an ion trap mass analyzer and the fragment mass accuracy was set to 0.8 Da. Considered modifications were methionine oxidation, deamidation (variable) and carbamidomethyl cysteine (fixed). Two missed cleavages for the enzyme were permitted. Peptides with a score less than 20 were filtered and proteins identified required bold red peptides. Protein identifications were checked manually and proteins with a Mascot score of 100 or higher with a minimum of two unique peptides from one protein having a *-b* or *-y* ion sequence tag of five residues or better were accepted.

**Results:**

Gel images were loaded into SameSpots software (TotalLabs) and analyzed individually (Figure 1). All saturated spots, dust spots, and noise were removed before gel normalization with manual verification that quality protein spots were not removed. SameSpots determined which gel was most similar to all of the other gels in the project and designated that gel as the reference gel to which all other images were matched. The log-standardized abundance was the variable subjected to statistical analysis. The standardized abundance was derived from the normalized spot volume (with background subtracted) and standardized against the intra-gel standard. The log of standardized abundance values were used so that data points approached a normal distribution around zero and were therefore suitable for statistical analysis. To examine protein expression differences between ΔgspD and M2, we used a Student’s t-test. The null hypothesis was that there was no change in protein expression and the average ratio (log-standardized abundance) between the two groups being compared was 1. Protein abundance was considered to be significantly different if the t-test p value was < 0.05.

Analysis with SameSpots revealed that 60 spots exhibited a statistically significant average change of at least 4-fold when comparing wild type vs M2*∆gspD*::kan (Table 2) samples. These spots were cored from the preparative gel (Figure 2), digested and run on the LTQ mass spectrometer to determine peptide composition. The resulting peptides from each spot were searched against *Acinetobacter* proteins in the NCBI database (3,487 sequences) using Mascot Daemon to identify candidate proteins responsible for expression changes. Proteins identifications from selected spots are included in Table 2.


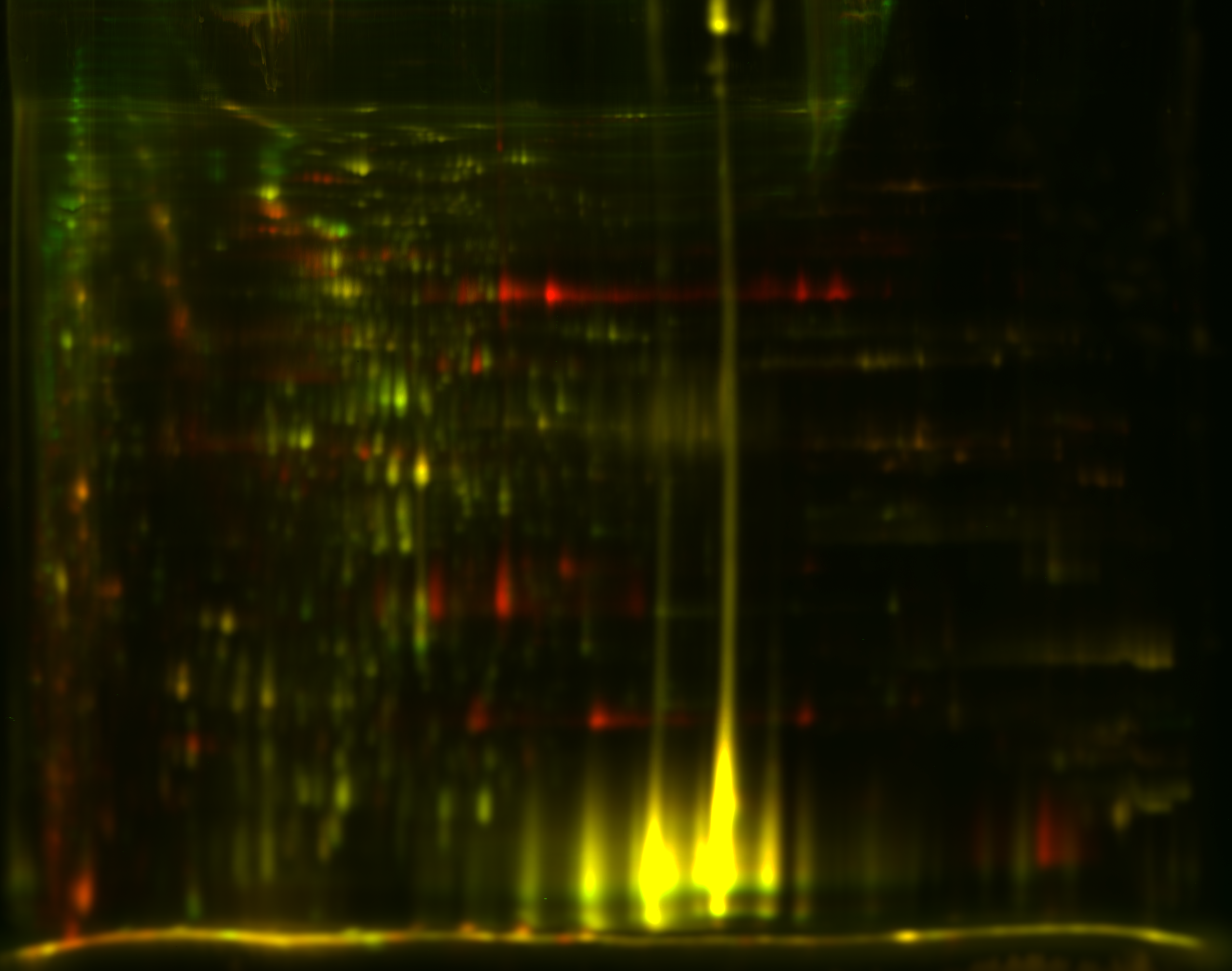


**Figure 1. Representative image from DIGE experiment.**  Image from gel #46641 (see Table 1) showing Cy3 (ΔgspD-2, green) and Cy5-labeled (M2-2, red) proteins that were isoelectric focused on pH strips (3-10), separated by size using SDS-PAGE, and visualized using a typhoon 9400 variable mode imager. A merged image of the Cy3 and Cy5-labeled proteins is shown. Proteins with greater abundance in the ΔgspD-2 sample appear green and proteins with greater abundance in the M2-2 sample appear red. Proteins that did not change relative abundance between the two samples appear yellow.

**Table 2. Protein Expression Changes in M2 vs ΔgspD**

| **Spot #** | **Desc** | **Mass** | **S^a^** | **M^b^** |
| --- | --- | --- | --- | --- |
| 81 | gi\|549999019\|ref\|WP_022575907.1\| hypothetical protein [Acinetobacter nosocomialis] | 162015 | 3049 | 47 |
|  | gi\|549995593\|ref\|WP_022575203.1\| hypothetical protein, partial [Acinetobacter nosocomialis] | 259729 | 378 | 5 |
|  | gi\|515189056\|ref\|WP_016805339.1\| ferrous iron transporter B [Acinetobacter nosocomialis] | 18586 | 186 | 3 |
| 284 | gi\|549999019\|ref\|WP_022575907.1\| hypothetical protein [Acinetobacter nosocomialis] | 162015 | 1790 | 28 |
|  | gi\|549996162\|ref\|WP_022575318.1\| surface adhesion protein [Acinetobacter nosocomialis] | 75056 | 969 | 16 |
|  | gi\|549995593\|ref\|WP_022575203.1\| hypothetical protein, partial [Acinetobacter nosocomialis] | 259729 | 218 | 3 |
| 291 | gi\|549996106\|ref\|WP_022575262.1\| membrane protein [Acinetobacter nosocomialis] | 88378 | 2217 | 34 |
|  | gi\|549999019\|ref\|WP_022575907.1\| hypothetical protein [Acinetobacter nosocomialis] | 162015 | 1436 | 25 |
|  | gi\|549997688\|ref\|WP_022575669.1\| TonB-denpendent receptor [Acinetobacter nosocomialis] | 73945 | 153 | 3 |
| 295 | gi\|549996106\|ref\|WP_022575262.1\| membrane protein [Acinetobacter nosocomialis] | 88378 | 2399 | 35 |
|  | gi\|549999019\|ref\|WP_022575907.1\| hypothetical protein [Acinetobacter nosocomialis] | 162015 | 1085 | 20 |
|  | gi\|487977529\|ref\|WP_002050327.1\| elongation factor G [Acinetobacter calcoaceticus/baumannii complex] | 79222 | 482 | 10 |
|  | gi\|549997688\|ref\|WP_022575669.1\| TonB-denpendent receptor [Acinetobacter nosocomialis] | 73945 | 377 | 7 |
| 298 | gi\|549996106\|ref\|WP_022575262.1\| membrane protein [Acinetobacter nosocomialis] | 88378 | 2428 | 34 |
|  | gi\|487977529\|ref\|WP_002050327.1\| elongation factor G [Acinetobacter calcoaceticus/baumannii complex] | 79222 | 1274 | 23 |
|  | gi\|549999019\|ref\|WP_022575907.1\| hypothetical protein [Acinetobacter nosocomialis] | 162015 | 885 | 16 |
|  | gi\|549987479\|ref\|WP_022574436.1\| hypothetical protein [Acinetobacter nosocomialis] | 219028 | 272 | 5 |
| 411 | gi\|549996105\|ref\|WP_022575261.1\| rhombotarget A [Acinetobacter nosocomialis] | 66196 | 1781 | 24 |
|  | gi\|549999019\|ref\|WP_022575907.1\| hypothetical protein [Acinetobacter nosocomialis] | 162015 | 1207 | 24 |
|  | gi\|549996714\|ref\|WP_022575401.1\| hypothetical protein [Acinetobacter nosocomialis] | 412376 | 413 | 7 |
|  | gi\|487977439\|ref\|WP_002050239.1\| 30S ribosomal protein S1 [Acinetobacter] | 61146 | 287 | 4 |
| 413 | gi\|549996105\|ref\|WP_022575261.1\| rhombotarget A [Acinetobacter nosocomialis] | 66196 | 1985 | 26 |
|  | gi\|549999019\|ref\|WP_022575907.1\| hypothetical protein [Acinetobacter nosocomialis] | 162015 | 1337 | 24 |
|  | gi\|549996714\|ref\|WP_022575401.1\| hypothetical protein [Acinetobacter nosocomialis] | 412376 | 744 | 12 |
|  | gi\|487977439\|ref\|WP_002050239.1\| 30S ribosomal protein S1 [Acinetobacter] | 61146 | 466 | 7 |
| 432 | gi\|491022852\|ref\|WP_004884546.1\| lytic transglycosylase [Acinetobacter calcoaceticus/baumannii complex] | 73182 | 1355 | 22 |
| 450 | gi\|549999051\|ref\|WP_022575939.1\| protein FilF [Acinetobacter nosocomialis] | 69237 | 1893 | 28 |
| 510 | gi\|549997266\|ref\|WP_022575561.1\| metallopeptidase [Acinetobacter nosocomialis] | 64494 | 2194 | 29 |
| 511 | gi\|549997266\|ref\|WP_022575561.1\| metallopeptidase [Acinetobacter nosocomialis] | 64494 | 1184 | 18 |
| 515 | gi\|549997266\|ref\|WP_022575561.1\| metallopeptidase [Acinetobacter nosocomialis] | 64494 | 2354 | 30 |
|  | gi\|490845969\|ref\|WP_004708032.1\| bifunctional purine biosynthesis protein purH [Acinetobacter nosocomialis] | 56388 | 1057 | 17 |
|  | gi\|549997086\|ref\|WP_022575536.1\| hypothetical protein [Acinetobacter nosocomialis] | 30350 | 658 | 11 |
|  | gi\|549999401\|ref\|WP_022576005.1\| UDP-N-acetylmuramoylalanyl-D-glutamate--2,6-diaminopimelate ligase [Acinetobacter nosocomialis] | 55274 | 346 | 6 |
|  | gi\|549997812\|ref\|WP_022575688.1\| sulfite reductase [Acinetobacter nosocomialis] | 62200 | 237 | 5 |
| 519 | gi\|549997266\|ref\|WP_022575561.1\| metallopeptidase [Acinetobacter nosocomialis] | 64494 | 2223 | 28 |
|  | gi\|490845969\|ref\|WP_004708032.1\| bifunctional purine biosynthesis protein purH [Acinetobacter nosocomialis] | 56388 | 116 | 2 |
| 522 | gi\|549997266\|ref\|WP_022575561.1\| metallopeptidase [Acinetobacter nosocomialis] | 64494 | 1662 | 24 |
|  | gi\|446575849\|ref\|WP_000653195.1\| hypothetical protein [Acinetobacter] | 18788 | 119 | 3 |
| 523 | gi\|549997266\|ref\|WP_022575561.1\| metallopeptidase [Acinetobacter nosocomialis] | 64494 | 1366 | 20 |
|  | gi\|490845969\|ref\|WP_004708032.1\| bifunctional purine biosynthesis protein purH [Acinetobacter nosocomialis] | 56388 | 419 | 7 |
| 560 | gi\|549997266\|ref\|WP_022575561.1\| metallopeptidase [Acinetobacter nosocomialis] | 64494 | 988 | 14 |
|  | gi\|549997086\|ref\|WP_022575536.1\| hypothetical protein [Acinetobacter nosocomialis] | 30350 | 580 | 9 |
|  | gi\|549999639\|ref\|WP_022576059.1\| phosphoglucosamine mutase [Acinetobacter nosocomialis] | 48434 | 452 | 8 |
| 662 | gi\|549996421\|ref\|WP_022575351.1\| alpha/beta hydrolase [Acinetobacter nosocomialis] | 51518 | 1874 | 23 |
|  | gi\|487976403\|ref\|WP_002049224.1\| serine hydroxymethyltransferase [Acinetobacter calcoaceticus/baumannii complex] | 45183 | 1077 | 17 |
|  | gi\|549996698\|ref\|WP_022575385.1\| cyclohexadienyl dehydrogenase [Acinetobacter nosocomialis] | 79990 | 282 | 5 |
|  | gi\|549997266\|ref\|WP_022575561.1\| metallopeptidase [Acinetobacter nosocomialis] | 64494 | 226 | 3 |
| 677 | gi\|549996421\|ref\|WP_022575351.1\| alpha/beta hydrolase [Acinetobacter nosocomialis] | 51518 | 1521 | 20 |
|  | gi\|491022534\|ref\|WP_004884231.1\| molecular chaperone DnaK [Acinetobacter calcoaceticus/baumannii complex] | 69366 | 529 | 11 |
|  | gi\|549996714\|ref\|WP_022575401.1\| hypothetical protein [Acinetobacter nosocomialis] | 412376 | 477 | 7 |
|  | gi\|488085619\|ref\|WP_002157016.1\| elongation factor Tu [Acinetobacter] | 43136 | 471 | 8 |
|  | gi\|490848809\|ref\|WP_004710872.1\| hypothetical protein [Acinetobacter nosocomialis] | 44710 | 341 | 6 |
|  | gi\|515184965\|ref\|WP_016803909.1\| long-chain fatty acid transporter [Acinetobacter nosocomialis] | 50613 | 204 | 3 |
|  | gi\|490846457\|ref\|WP_004708520.1\| enolase [Acinetobacter calcoaceticus/baumannii complex] | 46363 | 180 | 3 |
| 680 | gi\|549996421\|ref\|WP_022575351.1\| alpha/beta hydrolase [Acinetobacter nosocomialis] | 51518 | 217 | 3 |
|  | gi\|549999019\|ref\|WP_022575907.1\| hypothetical protein [Acinetobacter nosocomialis] | 162015 | 149 | 2 |
| 818 | gi\|549996729\|ref\|WP_022575416.1\| hypothetical protein [Acinetobacter nosocomialis] | 42213 | 995 | 16 |
|  | gi\|549997823\|ref\|WP_022575689.1\| membrane protein [Acinetobacter nosocomialis] | 38057 | 599 | 10 |
|  | gi\|487980900\|ref\|WP_002053629.1\| transaldolase [Acinetobacter calcoaceticus/baumannii complex] | 36420 | 527 | 10 |
|  | gi\|488085619\|ref\|WP_002157016.1\| elongation factor Tu [Acinetobacter] | 43136 | 212 | 4 |
|  | gi\|446971317\|ref\|WP_001048573.1\| malate--CoA ligase subunit beta [Acinetobacter] | 41675 | 187 | 3 |
|  | gi\|515963787\|ref\|WP_017394370.1\| fructose-1 6-bisphosphatase [Acinetobacter nosocomialis] | 37991 | 171 | 4 |
| 931 | gi\|549999384\|ref\|WP_022575988.1\| lipase [Acinetobacter nosocomialis] | 35080 | 877 | 14 |
|  | gi\|549999048\|ref\|WP_022575936.1\| esterase [Acinetobacter nosocomialis] | 31868 | 157 | 3 |
| 933 | gi\|549999384\|ref\|WP_022575988.1\| lipase [Acinetobacter nosocomialis] | 35080 | 1041 | 15 |
|  | gi\|487980200\|ref\|WP_002052939.1\| lytic murein transglycosylase B [Acinetobacter calcoaceticus/baumannii complex] | 36869 | 820 | 14 |
|  | gi\|487978581\|ref\|WP_002051365.1\| acetyl-CoA carboxylase carboxyl transferase subunit beta [Acinetobacter calcoaceticus/baumannii complex] | 33293 | 610 | 11 |
| 934 | gi\|487980200\|ref\|WP_002052939.1\| lytic murein transglycosylase B [Acinetobacter calcoaceticus/baumannii complex] | 36869 | 656 | 10 |
| 940 | gi\|549999019\|ref\|WP_022575907.1\| hypothetical protein [Acinetobacter nosocomialis] | 162015 | 797 | 15 |
|  | gi\|487980200\|ref\|WP_002052939.1\| lytic murein transglycosylase B [Acinetobacter calcoaceticus/baumannii complex] | 36869 | 678 | 10 |
|  | gi\|487978548\|ref\|WP_002051332.1\| succinyl-CoA synthetase subunit alpha [Acinetobacter calcoaceticus/baumannii complex] | 30873 | 235 | 4 |
|  | gi\|549999384\|ref\|WP_022575988.1\| lipase [Acinetobacter nosocomialis] | 35080 | 124 | 2 |
|  | gi\|549997823\|ref\|WP_022575689.1\| membrane protein [Acinetobacter nosocomialis] | 38057 | 122 | 2 |
|  | gi\|446575849\|ref\|WP_000653195.1\| hypothetical protein [Acinetobacter] | 18788 | 120 | 3 |
| 974 | gi\|549997086\|ref\|WP_022575536.1\| hypothetical protein [Acinetobacter nosocomialis] | 30350 | 1038 | 16 |
| 1006 | gi\|549999019\|ref\|WP_022575907.1\| hypothetical protein [Acinetobacter nosocomialis] | 162015 | 573 | 9 |
| 1048 | gi\|549999019\|ref\|WP_022575907.1\| hypothetical protein [Acinetobacter nosocomialis] | 162015 | 750 | 11 |
|  | gi\|549995593\|ref\|WP_022575203.1\| hypothetical protein, partial [Acinetobacter nosocomialis] | 259729 | 169 | 2 |
| 1071 | No ID |  |  |  |
| 1074 | gi\|549995593\|ref\|WP_022575203.1\| hypothetical protein, partial [Acinetobacter nosocomialis] | 259729 | 167 | 2 |
| 1076 | gi\|549987689\|ref\|WP_022574470.1\| hypothetical protein [Acinetobacter nosocomialis] | 28172 | 682 | 10 |
| 1105 | gi\|549999055\|ref\|WP_022575943.1\| protein FilA [Acinetobacter nosocomialis] | 27579 | 403 | 4 |
|  | gi\|515189821\|ref\|WP_016805510.1\| membrane protein [Acinetobacter nosocomialis] | 26411 | 220 | 3 |
|  | gi\|446575849\|ref\|WP_000653195.1\| hypothetical protein [Acinetobacter] | 18788 | 113 | 3 |
| 1142 | gi\|549999019\|ref\|WP_022575907.1\| hypothetical protein [Acinetobacter nosocomialis] | 162015 | 652 | 9 |
|  | gi\|549999055\|ref\|WP_022575943.1\| protein FilA [Acinetobacter nosocomialis] | 27579 | 238 | 3 |
|  | gi\|487979285\|ref\|WP_002052040.1\| membrane protein [Acinetobacter calcoaceticus/baumannii complex] | 22505 | 227 | 4 |
| 1173 | gi\|549999019\|ref\|WP_022575907.1\| hypothetical protein [Acinetobacter nosocomialis] | 162015 | 573 | 8 |
|  | gi\|446575849\|ref\|WP_000653195.1\| hypothetical protein [Acinetobacter] | 18788 | 417 | 7 |
|  | gi\|487981030\|ref\|WP_002053751.1\| membrane protein [Acinetobacter calcoaceticus/baumannii complex] | 27809 | 236 | 4 |
| 1229 | gi\|446575849\|ref\|WP_000653195.1\| hypothetical protein [Acinetobacter] | 18788 | 377 | 6 |
|  | gi\|487981030\|ref\|WP_002053751.1\| membrane protein [Acinetobacter calcoaceticus/baumannii complex] | 27809 | 312 | 5 |
| 1338 | gi\|549999019\|ref\|WP_022575907.1\| hypothetical protein [Acinetobacter nosocomialis] | 162015 | 812 | 11 |
|  | gi\|549995593\|ref\|WP_022575203.1\| hypothetical protein, partial [Acinetobacter nosocomialis] | 259729 | 153 | 2 |
| 1340 | gi\|549999019\|ref\|WP_022575907.1\| hypothetical protein [Acinetobacter nosocomialis] | 162015 | 815 | 11 |
| 1350 | gi\|549999019\|ref\|WP_022575907.1\| hypothetical protein [Acinetobacter nosocomialis] | 162015 | 1373 | 18 |
|  | gi\|549995593\|ref\|WP_022575203.1\| hypothetical protein, partial [Acinetobacter nosocomialis] | 259729 | 323 | 4 |
| 1362 | gi\|549999019\|ref\|WP_022575907.1\| hypothetical protein [Acinetobacter nosocomialis] | 162015 | 2372 | 35 |
| 1378 | gi\|549999019\|ref\|WP_022575907.1\| hypothetical protein [Acinetobacter nosocomialis] | 162015 | 1715 | 28 |
|  | gi\|549996729\|ref\|WP_022575416.1\| hypothetical protein [Acinetobacter nosocomialis] | 42213 | 236 | 5 |
|  | gi\|446575849\|ref\|WP_000653195.1\| hypothetical protein [Acinetobacter] | 18788 | 125 | 3 |
| 1379 | gi\|549999019\|ref\|WP_022575907.1\| hypothetical protein [Acinetobacter nosocomialis] | 162015 | 1611 | 26 |
|  | gi\|549996729\|ref\|WP_022575416.1\| hypothetical protein [Acinetobacter nosocomialis] | 42213 | 651 | 12 |
|  | gi\|446971317\|ref\|WP_001048573.1\| malate--CoA ligase subunit beta [Acinetobacter] | 41675 | 528 | 9 |
|  | gi\|446575849\|ref\|WP_000653195.1\| hypothetical protein [Acinetobacter] | 18788 | 142 | 3 |
| 1409 | gi\|487979285\|ref\|WP_002052040.1\| membrane protein [Acinetobacter calcoaceticus/baumannii complex] | 22505 | 323 | 6 |
|  | gi\|549999019\|ref\|WP_022575907.1\| hypothetical protein [Acinetobacter nosocomialis] | 162015 | 180 | 3 |
|  | gi\|446575849\|ref\|WP_000653195.1\| hypothetical protein [Acinetobacter] | 18788 | 106 | 2 |
| 1420 | gi\|549999019\|ref\|WP_022575907.1\| hypothetical protein [Acinetobacter nosocomialis] | 162015 | 256 | 4 |
| 1421 | gi\|549999019\|ref\|WP_022575907.1\| hypothetical protein [Acinetobacter nosocomialis] | 162015 | 210 | 4 |
| 1440 | gi\|549997266\|ref\|WP_022575561.1\| metallopeptidase [Acinetobacter nosocomialis] | 64494 | 2686 | 35 |
|  | gi\|549999411\|ref\|WP_022576015.1\| pyruvate dehydrogenase [Acinetobacter nosocomialis] | 63286 | 358 | 7 |
|  | gi\|549993398\|ref\|WP_022574858.1\| protein PaaC [Acinetobacter nosocomialis] | 57299 | 273 | 6 |
| 1447 | gi\|549999019\|ref\|WP_022575907.1\| hypothetical protein [Acinetobacter nosocomialis] | 162015 | 1632 | 28 |
|  | gi\|549997266\|ref\|WP_022575561.1\| metallopeptidase [Acinetobacter nosocomialis] | 64494 | 1315 | 16 |
|  | gi\|549999051\|ref\|WP_022575939.1\| protein FilF [Acinetobacter nosocomialis] | 69237 | 1243 | 18 |
|  | gi\|487975766\|ref\|WP_002048605.1\| molecular chaperone GroEL [Acinetobacter calcoaceticus/baumannii complex] | 57259 | 1103 | 17 |
|  | gi\|515186814\|ref\|WP_016804539.1\| acyl-CoA dehydrogenase [Acinetobacter nosocomialis] | 64940 | 604 | 11 |
|  | gi\|487980547\|ref\|WP_002053278.1\| trigger factor [Acinetobacter calcoaceticus/baumannii complex] | 49635 | 118 | 2 |
| 1449 | gi\|549999051\|ref\|WP_022575939.1\| protein FilF [Acinetobacter nosocomialis] | 69237 | 1610 | 24 |
|  | gi\|549999019\|ref\|WP_022575907.1\| hypothetical protein [Acinetobacter nosocomialis] | 162015 | 1266 | 22 |
|  | gi\|549997266\|ref\|WP_022575561.1\| metallopeptidase [Acinetobacter nosocomialis] | 64494 | 666 | 9 |
|  | gi\|487975766\|ref\|WP_002048605.1\| molecular chaperone GroEL [Acinetobacter calcoaceticus/baumannii complex] | 57259 | 296 | 5 |
| 1464 | gi\|549987689\|ref\|WP_022574470.1\| hypothetical protein [Acinetobacter nosocomialis] | 28172 | 726 | 10 |
|  | gi\|487978591\|ref\|WP_002051374.1\| hisitidine kinase [Acinetobacter calcoaceticus/baumannii complex] | 26763 | 566 | 9 |
|  | gi\|549997266\|ref\|WP_022575561.1\| metallopeptidase [Acinetobacter nosocomialis] | 64494 | 371 | 5 |
| 1505 | gi\|549997266\|ref\|WP_022575561.1\| metallopeptidase [Acinetobacter nosocomialis] | 64494 | 2298 | 30 |
|  | gi\|490845969\|ref\|WP_004708032.1\| bifunctional purine biosynthesis protein purH [Acinetobacter nosocomialis] | 56388 | 977 | 17 |
|  | gi\|549993010\|ref\|WP_022574784.1\| VGR [Acinetobacter nosocomialis] | 117217 | 156 | 4 |
| 1517 | gi\|549987689\|ref\|WP_022574470.1\| hypothetical protein [Acinetobacter nosocomialis] | 28172 | 1043 | 14 |
|  | gi\|549997266\|ref\|WP_022575561.1\| metallopeptidase [Acinetobacter nosocomialis] | 64494 | 733 | 10 |
| 1521 | gi\|549997086\|ref\|WP_022575536.1\| hypothetical protein [Acinetobacter nosocomialis] | 30350 | 1243 | 18 |
|  | gi\|518288148\|ref\|WP_019458356.1\| hypothetical protein [Acinetobacter] | 31846 | 937 | 14 |
|  | gi\|549986913\|ref\|WP_022574324.1\| glucose-1-phosphate thymidylyltransferase [Acinetobacter nosocomialis] | 33430 | 740 | 11 |
|  | gi\|549997823\|ref\|WP_022575689.1\| membrane protein [Acinetobacter nosocomialis] | 38057 | 638 | 10 |
|  | gi\|549987689\|ref\|WP_022574470.1\| hypothetical protein [Acinetobacter nosocomialis] | 28172 | 433 | 6 |
|  | gi\|549997266\|ref\|WP_022575561.1\| metallopeptidase [Acinetobacter nosocomialis] | 64494 | 333 | 5 |
| 1527 | gi\|549999019\|ref\|WP_022575907.1\| hypothetical protein [Acinetobacter nosocomialis] | 162015 | 634 | 9 |
|  | gi\|446575849\|ref\|WP_000653195.1\| hypothetical protein [Acinetobacter] | 18788 | 176 | 4 |
| 1553 | gi\|549997116\|ref\|WP_022575539.1\| chorismate mutase [Acinetobacter nosocomialis] | 21236 | 430 | 8 |
|  | gi\|549999019\|ref\|WP_022575907.1\| hypothetical protein [Acinetobacter nosocomialis] | 162015 | 113 | 2 |
| 1589 | gi\|549997266\|ref\|WP_022575561.1\| metallopeptidase [Acinetobacter nosocomialis] | 64494 | 1795 | 27 |
| 1594 | gi\|549997086\|ref\|WP_022575536.1\| hypothetical protein [Acinetobacter nosocomialis] | 30350 | 1199 | 17 |
|  | gi\|447152590\|ref\|WP_001229846.1\| 5,10-methylene-tetrahydrofolate dehydrogenase [Acinetobacter] | 29911 | 544 | 9 |
|  | gi\|549999987\|ref\|WP_022576125.1\| dihydrodipicolinate reductase [Acinetobacter nosocomialis] | 28857 | 229 | 4 |
|  | gi\|549997266\|ref\|WP_022575561.1\| metallopeptidase [Acinetobacter nosocomialis] | 64494 | 194 | 4 |
|  | gi\|446997106\|ref\|WP_001074362.1\| cell division inhibitor MinD [Acinetobacter] | 30163 | 185 | 3 |
| 1595 | gi\|549997086\|ref\|WP_022575536.1\| hypothetical protein [Acinetobacter nosocomialis] | 30350 | 1141 | 18 |
|  | gi\|487981864\|ref\|WP_002054567.1\| cysteine synthase [Acinetobacter calcoaceticus/baumannii complex] | 34009 | 523 | 10 |
|  | gi\|447152590\|ref\|WP_001229846.1\| 5,10-methylene-tetrahydrofolate dehydrogenase [Acinetobacter] | 29911 | 238 | 5 |
|  | gi\|446997106\|ref\|WP_001074362.1\| cell division inhibitor MinD [Acinetobacter] | 30163 | 212 | 4 |
|  | gi\|549999048\|ref\|WP_022575936.1\| esterase [Acinetobacter nosocomialis] | 31868 | 159 | 3 |
| 1626 | gi\|549996421\|ref\|WP_022575351.1\| alpha/beta hydrolase [Acinetobacter nosocomialis] | 51518 | 1266 | 19 |
|  | gi\|549997086\|ref\|WP_022575536.1\| hypothetical protein [Acinetobacter nosocomialis] | 30350 | 276 | 4 |
| 1649 | No ID |  |  |  |
| 1652 | gi\|549999019\|ref\|WP_022575907.1\| hypothetical protein [Acinetobacter nosocomialis] | 162015 | 2598 | 43 |
|  | gi\|549996105\|ref\|WP_022575261.1\| rhombotarget A [Acinetobacter nosocomialis] | 66196 | 248 | 5 |
|  | gi\|491022534\|ref\|WP_004884231.1\| molecular chaperone DnaK [Acinetobacter calcoaceticus/baumannii complex] | 69366 | 128 | 3 |
| 1655 | gi\|549999019\|ref\|WP_022575907.1\| hypothetical protein [Acinetobacter nosocomialis] | 162015 | 2464 | 38 |
|  | gi\|549995593\|ref\|WP_022575203.1\| hypothetical protein, partial [Acinetobacter nosocomialis] | 259729 | 405 | 5 |
|  | gi\|515189056\|ref\|WP_016805339.1\| ferrous iron transporter B [Acinetobacter nosocomialis] | 18586 | 215 | 3 |
| 1657 | gi\|549997266\|ref\|WP_022575561.1\| metallopeptidase [Acinetobacter nosocomialis] | 64494 | 1712 | 24 |
|  | gi\|490845969\|ref\|WP_004708032.1\| bifunctional purine biosynthesis protein purH [Acinetobacter nosocomialis] | 56388 | 651 | 11 |
|  | gi\|549999019\|ref\|WP_022575907.1\| hypothetical protein [Acinetobacter nosocomialis] | 162015 | 463 | 8 |
|  | gi\|549998113\|ref\|WP_022575719.1\| glutamyl-tRNA(Gln) amidotransferase [Acinetobacter nosocomialis] | 53063 | 309 | 6 |
|  | gi\|487977439\|ref\|WP_002050239.1\| 30S ribosomal protein S1 [Acinetobacter] | 61146 | 280 | 5 |

*a* The protein score is derived from Mascot and provides an indication of how well the peptides matched the indicated protein sequence. The actual score is calculated by the following equation: protein score = -10*Log(P), where P is the probability that the protein match is a random event. Scores above 100 indicate that p < 0.05.

*b* The protein match score indicates the number of unique peptides that matched the sequence of the identified protein. Two unique peptide matches to a protein sequence confirms the identity of a protein.
