# Supplementary material for: Medically Relevant Acinetobacter Species Require a Type II Secretion System and Specific Membrane-Associated Chaperones for the Export of Multiple Substrates and Full Virulence
Source: PLoS Pathog. 2016 Jan 14;12(1):e1005391. doi: 10.1371/journal.ppat.1005391 (PMC4713064; doi:10.1371/journal.ppat.1005391)
Supplement: S1 Table — (DOCX) [file ppat.1005391.s002.docx]

| **S1 Table. Plasmids and bacterial strains included in the study** | | |
| --- | --- | --- |
| **Plasmid or strain** | **Relevant characteristic(s)** | **Reference/**  **Source** |
| **Plasmids** |  |  |
| pKD13 | Contains kanamycin resistance gene from Tn5 flanked by FRT sites | ([1](#_ENREF_1)) |
| pFLP2 | Encodes Flp recombinase | ([2](#_ENREF_2)) |
| pGEM-T-Ez | General cloning plasmid | Promega |
| pGEM-*gspD*::kan | pGEM containing a *gspD*::kan with 1kb flanking DNA up and downstream | This study |
| pGEM-*lipA*::kan | pGEM containing *lipA*::kan with 1kb flanking DNA up and downstream | This study |
| pGEM-*abhA*::kan | pGEM containing *abhA*::kan with 1kb flanking DNA up and downstream | This study |
| pRSM4063 | pSMART-LCkan containing an empty mTn*7* element from pRSM3510 along with 2kb flanking DNA up and downstream of the *att*Tn*7* site in *A. nosocomialis* M2 | ([3](#_ENREF_3)) |
| pRSM4063-*gspNCD* | pRSM4063 containing the *gspNCD* locus with the predicted *gspN* promoter | This study |
| pRSM4063-*gspN^P^-gspD* | pRSM4063-*gspNCD* lacking *gspNC* | This study |
| pWH1266 | *Acinetobacter-E. coli* shuttle vector | ([4](#_ENREF_4)) |
| pWH-*cpaA*-*his* | pWH1266 containing *cpaA* along with its predicted native promoter | This study |
| pWH-*cpaA*-*his* | pWH-*cpaA* containing a hexa-histidine tag on the carboxy terminus of *cpaA* | This study |
| pWH-*cpaA-cpaB* | pWH1266 containing *cpaA* and *cpaB* along with the predicted native promoter | This study |
| pWH-*cpaA-his-cpaB* | pWH-*cpaA-cpaB* containing a hexa-histidine tag on the C-terminus of *cpaA* | This study |
| pWH-*lipA* | pWH1266 containing *lipA* along with the predicted promoter | This study |
| pWH-*lipA-his* | pWH-*lipA* containing a hexa-histidine tag on the carboxy terminus of *lipA* | This study |
| pWH-*lipB-lipA* | pWH1266 containing *lipB* and *lipA* along with the predicted native promoter | This study |
| pWH-*lipB-lipA-his* | pWH-*lipB-lipA* containing a hexa-histidine tag on the carboxy terminus of *lipA* | This study |
| pWH-*abhA* | pWH1266 containing *abhA* along with its predicted native promoter | This study |
| pWH-*abhA-his* | pWH-*abhA* containing a hexa-histidine tag on the carboxy terminus of *abhA* | This study |
| pWH-*gspFG* | pWH1266 containing the *gspFG* locus and the predicted native promoter | This study |
| pWH-*gspF*^P^-*gspG* | pWH-*gspFG* with an in-frame deletion of the *gpsF* gene | This study |
| pWH-*gspF*^P^-*gspG-FLAG* | pWH-*gspF*^P^-*gspG* containing a C-terminal FLAG on *gspG* | This study |
| pRSM4063-*lipA* | pRSM4063 containing the *lipA* gene and its predicted native promoter | This study |
| pRSM4063-*abhA* | pRSM4063 containing the *abhA* gene and its predicted native promoter | This study |
| **Strains** |  |  |
| *A. nosocomialis* strain M2 | Metro Health Systems Clinical Isolate | ([5](#_ENREF_5), [6](#_ENREF_6)) |
| M2∆*gspD*::kan | *A. nosocomialis* strain M2 containing a deletion of *gspD* and replacement with a kanamycin resistance cassette | This study |
| M2∆*gspD*::kan (*gspD*+) | M2∆*gspD*::kan with a mini-Tn*7* element containing the *gspD* gene driven off the predicted *gspN* promoter | This study |
| M2∆*gspD*::kan-*sacB* | *A. nosocomialis* strain M2 containing a deletion of *gspD* and replacement with a kanamycin resistance cassette and the *sacB* gene | This study |
| M2∆*gspD*::frt | *A. nosocomialis* strain M2 containing an in frame, deletion of *gspD* | This study |
| M2∆*gspD*::frt (*gspD*+) | M2∆*gspD*::frt with a mini-Tn*7* element containing the *gspD* gene driven off the predicted *gspN* promoter | This study |
| M2∆*abhA*::kan | *A. nosocomialis* strain M2 containing a deletion of *abhA* and replacement with a kanamycin resistance cassette | This study |
| M2∆*abhA*::kan (*abhA*+) | M2∆*abhA*::kan with a mini-Tn*7* element containing *abhA* driven off the predicted *abhA* promoter | This study |
| M2∆*lipA*::kan | *A. nosocomialis* strain M2 containing a deletion of *lipA* and replacement with a kanamycin resistance cassette | This study |
| M2∆*lipA*::kan (*lipA*+) | M2∆*lipA*::kan with a mini-Tn*7* element containing *lipA* driven off the predicted *lipA* promoter | This study |
| M2∆*pilA*::frt | *A. nosocomialis* strain M2 containing an in frame deletion of *pilA* and replacement with FLP scar | ([3](#_ENREF_3)) |
| M2∆*pilD*::kan | *A. nosocomialis* strain M2 containing a deletion of *pilD* and replacement with a kanamycin resistance cassette | ([7](#_ENREF_7)) |
| M2∆*pilD*::kan (*pilD*+) | M2∆*pilD* with a mini-Tn7 element containing the *pilD* gene transcribed from its predicted promoter | ([7](#_ENREF_7)) |
| *A. baumannii* ATCC 17978 | Type strain | ATCC |
| *A. baumannii* ATCC 19606 | Type strain | ATCC |
| *A. pittii* 31132 | Clinical isolate | Nationwide Children’s Hospital |
| *A. pittii* 31357 | Clinical isolate | Nationwide Children’s Hospital |
| *Acinetobacter* spp. 33904 | Clinical isolate | Nationwide Children’s Hospital |
| *E. coli* DH5a | General cloning strain | Invitrogen |
| *E. coli* EC100D | General cloning strain, *pir*^+^ | Epicentre |
| *E. coli* TOP10 | General cloning strain | Invitrogen |
| *E. coli* HB101(pRK2013) | Conjugation helper strain | ([8](#_ENREF_8)) |

**SI Table References:**

1. Datsenko KA, Wanner BL. One-step inactivation of chromosomal genes in *Escherichia coli* K-12 using PCR products. Proceedings of the National Academy of Sciences of the United States of America. 2000;97(12):6640-5.

2. Kumar A, Dalton C, Cortez-Cordova J, Schweizer HP. Mini-Tn7 vectors as genetic tools for single copy gene cloning in *Acinetobacter baumannii*. Journal of microbiological methods. 2010;82(3):296-300.

3. Harding CM, Nasr MA, Kinsella RL, Scott NE, Foster LJ, Weber BS, et al. *Acinetobacter* strains carry two functional oligosaccharyltransferases, one devoted exclusively to type IV pilin, and the other one dedicated to *O*-glycosylation of multiple proteins. Molecular microbiology. 2015;96(5):1023-41.

4. Hunger M, Schmucker R, Kishan V, Hillen W. Analysis and nucleotide sequence of an origin of DNA replication in *Acinetobacter calcoaceticus* and its use for *Escherichia coli* shuttle plasmids. Gene. 1990;87(1):45-51.

5. Niu C, Clemmer KM, Bonomo RA, Rather PN. Isolation and characterization of an autoinducer synthase from *Acinetobacter baumannii*. Journal of Bacteriology. 2008;190(9):3386-92.

6. Carruthers MD, Harding CM, Baker BD, Bonomo RA, Hujer KM, Rather PN, et al. Draft Genome Sequence of the Clinical Isolate *Acinetobacter nosocomialis* Strain M2. Genome Announcements. 2013;1(6).

7. Harding CM, Tracy EN, Carruthers MD, Rather PN, Actis LA, Munson RS, Jr. *Acinetobacter baumannii* strain M2 produces type IV pili which play a role in natural transformation and twitching motility but not surface-associated motility. mBio. 2013;4(4).

8. Figurski DH, Helinski DR. Replication of an origin-containing derivative of plasmid RK2 dependent on a plasmid function provided in trans. Proceedings of the National Academy of Sciences of the United States of America. 1979;76(4):1648-52.
