# Supplementary figures and images for: Medically Relevant Acinetobacter Species Require a Type II Secretion System and Specific Membrane-Associated Chaperones for the Export of Multiple Substrates and Full Virulence
Source: PLoS Pathog. 2016 Jan 14;12(1):e1005391. doi: 10.1371/journal.ppat.1005391 (PMC4713064; doi:10.1371/journal.ppat.1005391)

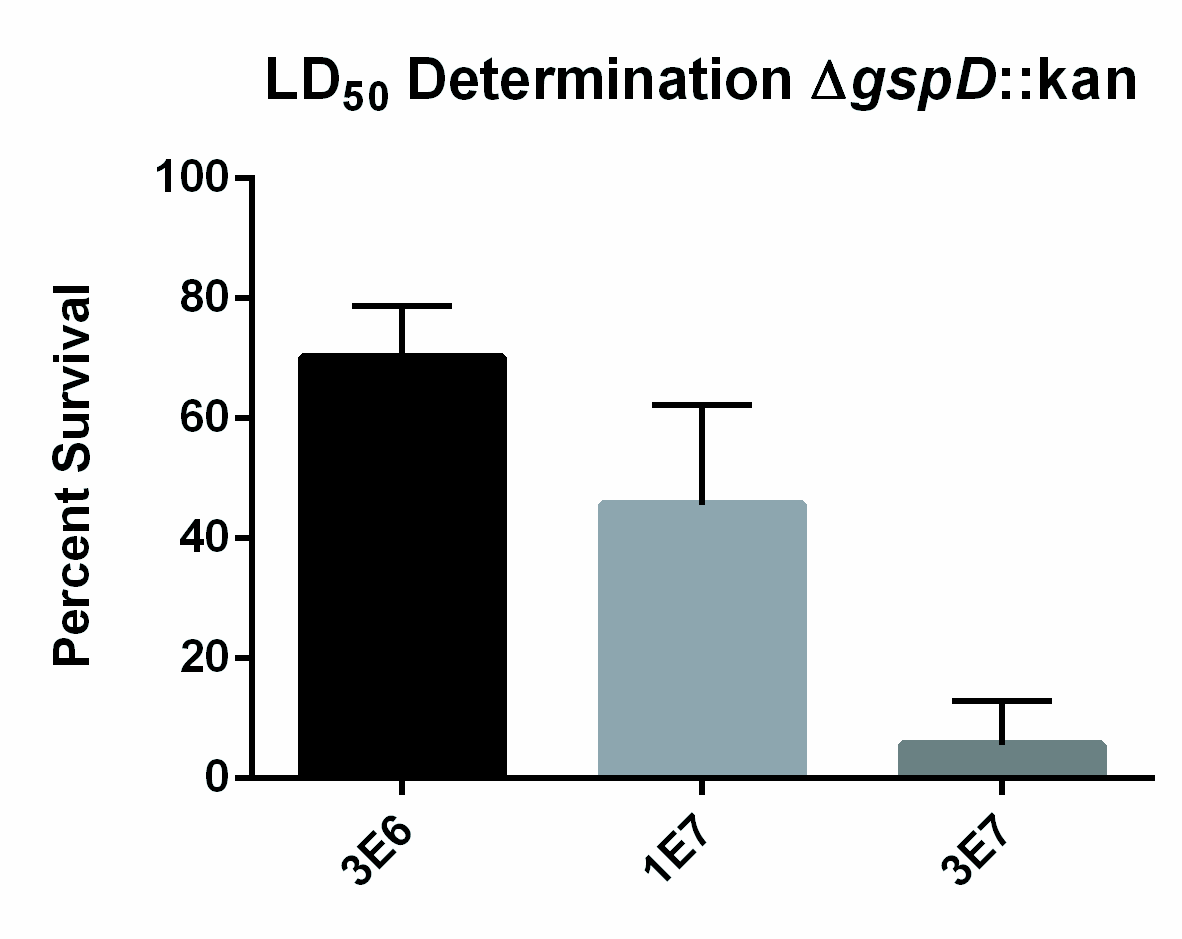

Supplement: S2 Fig — Groups of 10 G. mellonella were injected with 10μL M2∆ gspD::kan mutant at 3X106, 1X107, or 3X107 CFUs. Eighteen hours after injection larvae were checked for viability as determined by melanin accumulation and motility. (TIF) [file ppat.1005391.s005.tif]

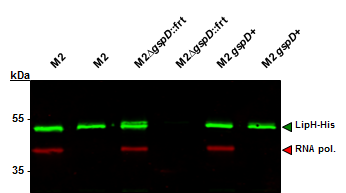

Supplement: S3 Fig — Western blot analysis on whole cell lysates and secreted protein fractions probing for LipH-His. All strains and fractions were also analyzed for RNA polymerase expression, which served as a lysis control. LipH-His expression was detected in all strains carrying the pWH-lipH-his; however, LipH-His secretion was only detected in the parental M2 strain and the complemented gspD::frt strain, but not the ∆ gspD::frt strain. (TIF) [file ppat.1005391.s006.tif]

**Survival**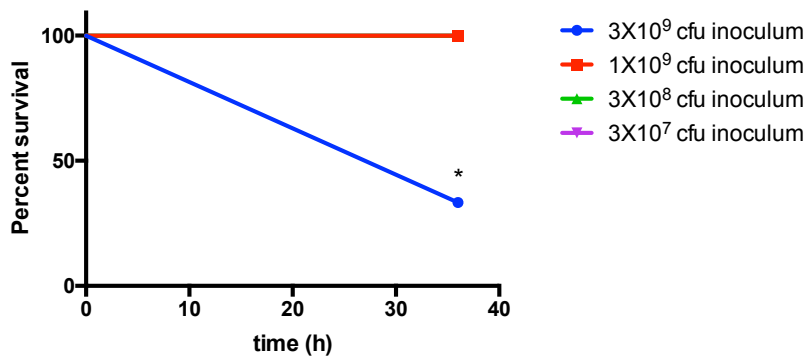**Lung**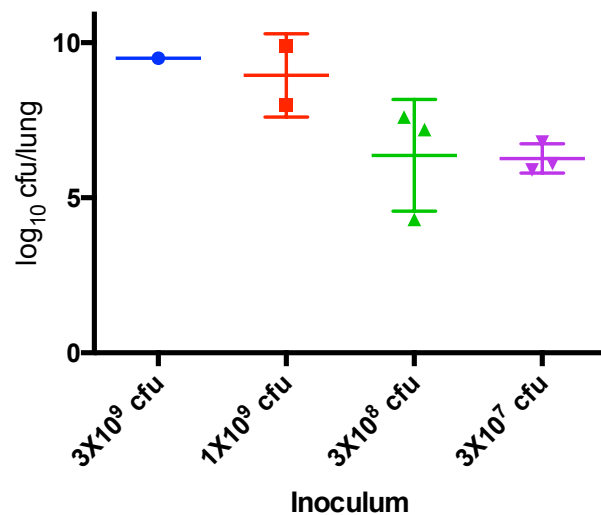**Liver**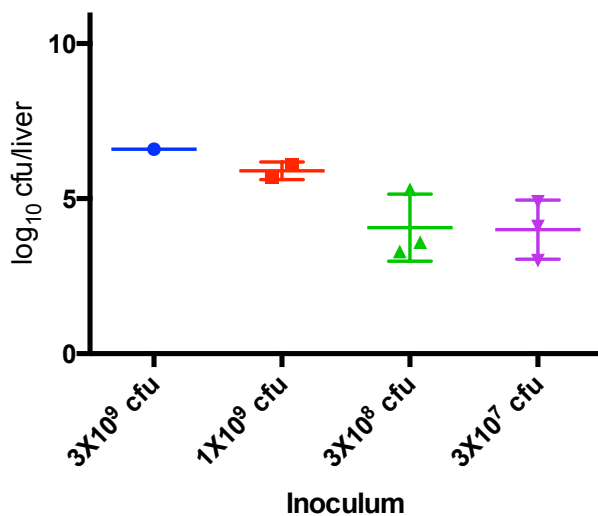**Spleen**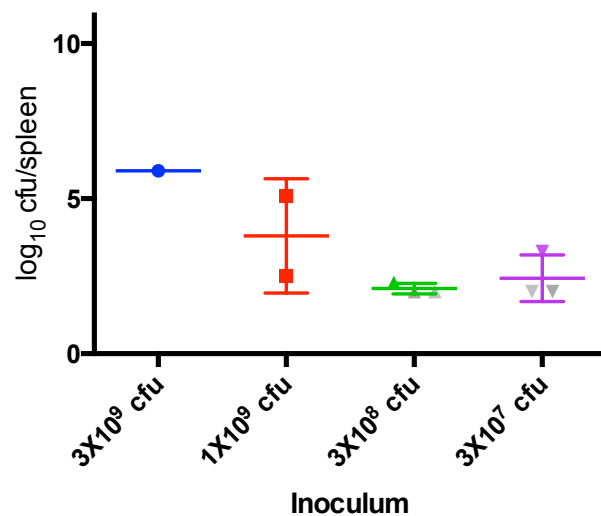

Supplement: S4 Fig — Four groups of three mice were intranasally inoculated with either 3X107, 3X108, 1X109, or 3X109 CFU of A. nosocomialis strain M2. Thirty six hours post infection surviving mice were sacrificed and organs were harvested for CFU enumeration. A single mouse from the 1X109 CFU dose group had to be removed post anesthesia and was excluded from this analysis. (PDF) [file ppat.1005391.s007.pdf]
